# Self-hydrogenated shell promoting photocatalytic H_2_ evolution on anatase TiO_2_

**DOI:** 10.1038/s41467-018-05144-1

**Published:** 2018-07-16

**Authors:** Yue Lu, Wen-Jin Yin, Kai-Lin Peng, Kuan Wang, Qi Hu, Annabella Selloni, Fu-Rong Chen, Li-Min Liu, Man-Ling Sui

**Affiliations:** 1 Institute of Microstructure and Properties of Advanced Materials, Beijing University of Technology, Beijing, 100124 China; 2 Beijing Computational Science Research Center, Beijing, 100084 China; 3 Department of Engineering and System Science, National Tsing Hua University, No. 101, Section 2, Kuang-Fu Road, Hsinchu, 30013 Taiwan China; 4 Department of Chemistry, Princeton University, Princeton, New Jersey 08544 United States; 5 Department of Materials Science and Engineering, City University, Hong Kong, China; 6 School of Physics, Beihang University, Beijing, 1000834 China

## Abstract

As one of the most important photocatalysts, TiO_2_ has triggered broad interest and intensive studies for decades. Observation of the interfacial reactions between water and TiO_2_ at microscopic scale can provide key insight into the mechanisms of photocatalytic processes. Currently, experimental methodologies for characterizing photocatalytic reactions of anatase TiO_2_ are mostly confined to water vapor or single molecule chemistry. Here, we investigate the photocatalytic reaction of anatase TiO_2_ nanoparticles in water using liquid environmental transmission electron microscopy. A self-hydrogenated shell is observed on the TiO_2_ surface before the generation of hydrogen bubbles. First-principles calculations suggest that this shell is formed through subsurface diffusion of photo-reduced water protons generated at the aqueous TiO_2_ interface, which promotes photocatalytic hydrogen evolution by reducing the activation barrier for H_2_ (H–H bond) formation. Experiments confirm that the self-hydrogenated shell contains reduced titanium ions, and its thickness can increase to several nanometers with increasing UV illuminance.

## Abstract (web summary)

Photocatalytic water splitting on TiO_2_ is a promising route to H_2_ fuel production, but the mechanistic pathway at the water–TiO_2_ interface remains poorly understood. Here, using liquid environmental TEM and first-principles calculations, the authors unveil the formation of a self-hydrogenated shell on the TiO_2_ surface that further promotes H_2_ production.

## Introduction

Understanding the reaction pathways of photocatalytic hydrogen evolution at the water/TiO_2_ interface is of crucial importance for developing clean renewable energy technologies^1–15^. This understanding can be greatly enhanced by direct observation of the interfacial reactions on TiO_2_ at the nanometer or even atomic scale^4–7^. Scanning tunneling microscopy (STM)^5,6,8^ and environmental transmission electron microscopy (ETEM)^7^ have proven to be powerful tools for this purpose. For example, previous STM studies have shown that submonolayer water and individual water molecules dissociate at oxygen vacancies on TiO_2_ surfaces^6^. Recent STM and surface X-ray diffraction measurements have revealed that the structure of water-dipped rutile TiO_2_ consists of a (2 × 1) ordered array of hydroxyl molecules with molecular water in the second layer^4^. Using an ETEM equipped with water vapor flow and UV illumination system, Zhang et al. found that a heavily hydroxylated amorphous layer of one or two atomic plane thickness covered the anatase TiO_2_ surface during UV light irradiation in water vapor^7^. However, only a limited amount of water is allowed to operate in STM and ETEM, and it is thus difficult to uncover the photocatalytic reaction pathways at the liquid H_2_O/TiO_2_ interface, especially those occurring in real aqueous environment.

Here, we employ a liquid environmental transmission electron microscopy (LETEM)^16–18^ to investigate the photocatalytic reactions occurring on the surface of anatase TiO_2_ nanoparticles (NPs) immersed in water under ultraviolet (UV) illumination. The photocatalytic reactions found in this research are very different from those observed under vapor conditions in the ETEM^7^. In water environment, we observe the natural growth of a nanoscale shell on the surface of the anatase NPs, followed by the generation of hydrogen nanobubbles. Using electron energy loss spectroscopy (EELS), we find that this shell contains reduced Ti ions and transforms to crystalline reduced titanium oxide (Ti_2_O_3_ or TiO) after drying in air. First-principle calculations allow us to rationalize these findings by showing that hydrogen atoms resulting from reaction of water protons with photoexcited electrons on the TiO_2_ surface can easily migrate subsurface. This leads to formation of a metastable hydrogenated shell containing reduced Ti^3+^ ions, which reduces the activation energy of H_2_ evolution. The nanoscale hydrogenated TiO_2_ shell is also observed during photocatalytic reaction on TiO_2_ NPs loaded with Pt co-catalyst, even though the kinetics of photocatalysis in the presence of co-catalyst is much faster than in the case of neat TiO_2_ NPs. Our work reveals that the formation of a nanoscale hydrogenated TiO_2_ shell is vital for the generation of hydrogen bubbles during the photocatalytic process, thus providing important insight into the fundamental mechanism of photocatalytic hydrogen generation on anatase TiO_2_.

## Results

### Low dose TEM observation of photocatalytic water splitting on TiO_2_

**Fig. 1:**
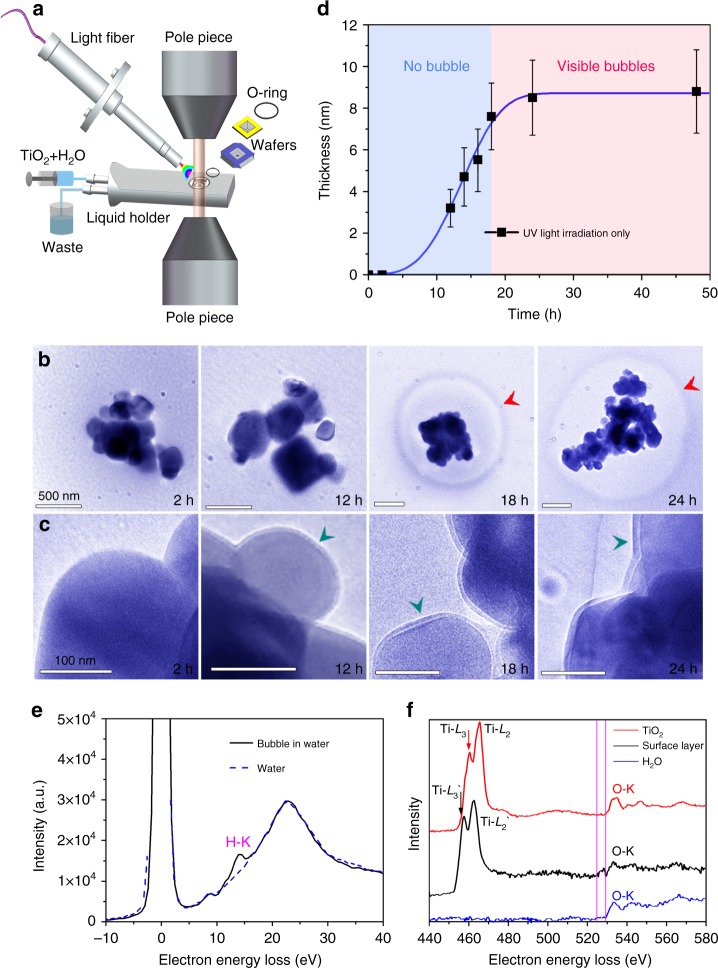
TEM/EELS analyses of surface shell growth on anatase TiO_2_ and hydrogen nanobubble formation after UV light illumination. **a** Experimental setup of a fluidic TEM holder with function of in situ UV illumination (see details in Methods). Water-immersed TiO_2_ NPs are injected into the liquid chamber of the fluidic holder for TEM imaging. The UV illumination area covers the whole liquid chamber, which is about 3 mm. **b** Photocatalysis experiments with different times of UV exposure. Each TEM image is recorded independently with fresh aqueous suspensions of TiO_2_ NPs controlled by the fluidic holder. Red arrow indicates the gas bubble around the TiO_2_ NPs. **c** Magnified views of the TEM images in **b**. After 12 h of UV illumination, a surface shell of 3.2 nm (indicated by a green arrow) covering the anatase TiO_2_ NPs is observed. **d** Thickness of the surface shell on anatase TiO_2_ vs. UV illumination time: experimental data (black squares) and fitted curve based on the KJMA equation^21^ (blue line). Growth of the surface shell abruptly decreases when the bubbles are visible at ~18 h (reddish area). **e** EELS spectra from the bubble (black line) and from the pure water region (dashed blue line). **f** EELS for the initial crystalline surface of TiO_2_ (red curve), the surrounding water (blue curve), and the surface shell (black curve). Purple vertical lines highlight the pre-edge feature at ~527 eV, before the O-K edge in the spectrum of surface shell

Anatase TiO_2_ NPs were immersed in water as 0.1 mol L^–1^ aqueous suspension and injected into the LETEM through a homemade liquid flow holder (Fig. 1a)^16–18^. An UV light fiber is introduced in between the pole pieces inside the LETEM, which releases a UV source (characteristic wavelength of 254, 297, 315, 335, 365, 404, and 425 nm) with a flux of 100 mW cm^−2^ at room temperature (Fig. 1a and Methods). The anatase TiO_2_ samples can be then illuminated in situ by the UV light to investigate the photocatalytic reaction on aqueous TiO_2_. In order to minimize the radiolytic effect of the electron beam on liquid water^19^ and metal oxide NPs^20^, we refreshed the TiO_2_ NPs solution for each UV exposure experiment via the fluidic holder, e.g., we repeated the photocatalytic reaction for different lengths of UV illumination using a new TiO_2_ NPs solution. Furthermore, we turned off the electron beam during the UV light illumination process and each TEM frame was recorded at low electron dose rate of about 3 e^−^·Å^−2^ s^−1^ for 1 s. The TEM images in Fig. 1b, c show the gradual growth of bubbles (red arrow in Fig. 1b) around the TiO_2_ NPs and the formation of surface shells (green arrows in Fig. 1c) on the TiO_2_ NPs. The bubbles were not visible around the NPs until 18 h of UV illumination. It is important to emphasize that, no matter whether TiO_2_ NPs are present or not, the bubbles observed here do not come from the radiolytic dissociation of water molecules by electron beam irradiation during the LETEM observations (see detailed discussion in Supplementary Notes 1 and 2, Supplementary Figs. 1 and 2).

For longer times, the size of the bubble increases steadily: the projection area is 2.6 μm^2^ at 18 h and becomes 4.7 μm^2^ at 24 h. The data presented as black squares in Fig. 1d show the thickness of the shell on the TiO_2_ NPs vs. UV illumination time *t*. These data are fitted (blue curve in Fig. 1d) with a KJMA equation^21^ *f* = 1–exp(–*kt*^*n*^), where *f* is the thickness normalized to the maximum thickness of the shell. The *n* value is ~3.4, which suggests that the growth mode of the surface shell is a 2D growth toward the water/TiO_2_ interface. The derivative of the curve in Fig. 1d shows that the growth rate of the shell increases up to ~0.7 nm/h in the initial stage, but abruptly drops to less than 0.1 nm/h at when the bubbles start to appear (*t* ~ 18 h). Finally, the thickness of the shell reaches a maximum of about 8.8 ± 2.0 nm. The long incubation time of 16–19 h for the photocatalytic generation of hydrogen at the macroscopic scale has also been confirmed by the detection of the amount of gas evolution in the pure anatase TiO_2_ aqueous solution in an independent experiment carried out outside the electron microscope (Supplementary Fig. 3a).

### Analysis of hydrogenated TiO_2_ shell

In order to understand the formation mechanism of the surface shell on TiO_2_ and the generation of nanobubbles, we analyzed the chemical composition of the bubbles and surface shell after each stage of the photocatalytic process with EELS (see Methods). In the spectrum acquired from the area of the bubbles (black curve in Fig. 1e), a peak presents at ~13 eV, which confirms that the bubbles are composed of hydrogen^22^. On the other hand, neither apparent hydrogen signal (dashed blue curve in Fig. 1e) nor bubbles (Supplementary Fig. 1b) were detected for pure water under electron beam irradiation. Fig. 1f shows EELS spectra for anatase TiO_2_ (red curve), water (blue curve) and the surface shell (black curve), respectively. The spectrum from the surface shell shows a shift (~2.7 eV) to lower energies and a drop of the integrated intensity ratio of the Ti–*L*_2_/*L*_3_ peaks as compared to the spectrum from anatase TiO_2_. This indicates a valence state reduction of titanium in the surface shell^23,24^. It is noteworthy that the O K edge of the surface shell has a pre-edge at ~527 eV (between the purple vertical lines in Fig. 1f), which is different from the regular K-edge of oxygen in H_2_O and TiO_2_. The same pre-edge of oxygen has been detected in studies of Al(OH)_3_^25^, and has been attributed to unpaired O originating from the loss of hydrogen in the hydroxyl layer of Al(OH)_3_^26^ induced by electron radiolysis. This provides implicit evidence that the surface shell around TiO_2_ NPs also contains hydroxyl species due to diffusion of H atoms into the anatase TiO_2_ NPs.

### Formation mechanism of hydrogenated TiO_2_ shell

**Fig. 2:**
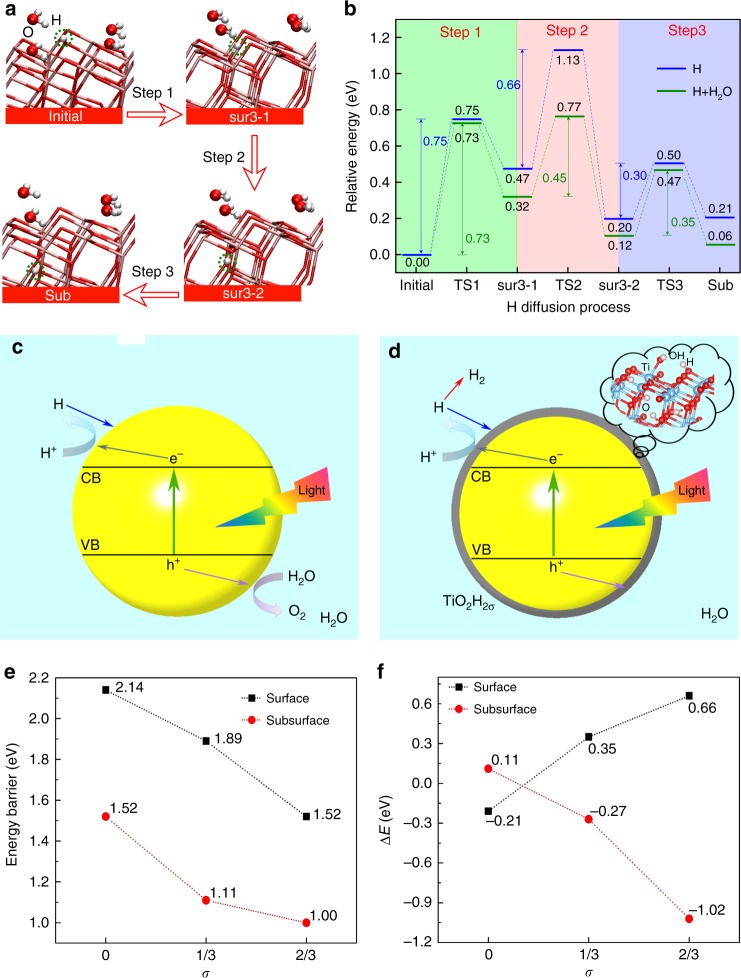
Formation mechanism and reactivity of the hydrogenated TiO_2_ shell. **a** Atomic structures of four key intermediates—initial state, sur3-1 state, sur3-2 state, and sub state—in the surface subsurface diffusion of a H atom. In the sur3-1 (sur3-2) state, atomic hydrogen is adsorbed on a surface three-fold O with the hydrogen pointing toward the vacuum (toward the bulk). In the sub (or final) state the hydrogen is adsorbed at a subsurface O atom. The O atom is red, H is gray blue, and the pink-red sticks represent the TiO_2_ system. Red arrows indicate the three steps (Steps 1–3) for the diffusion of H (marked by the green dot circle) into TiO_2_. **b** Potential energy profile for the surface subsurface diffusion of atomic hydrogen. Energies are relative to the initial state, and ‘TS’ indicates transition states. Blue and green bars represent energies in the absence and presence of adsorbed water, respectively. **c** Schematic representation of the photocatalytic process: under UV light, the photoexcited electron–hole pairs can split water to form hydrogen and oxygen. **d** Modified photocatalytic process described in this work: the hydrogen atoms diffuse first into TiO_2_ (blue arrow) to form the hydrogenated TiO_2_ shell on the NP^14,15^ before they are desorbed into water to form the hydrogen bubble. **e**, **f** Energy barriers and the desorption energies for the process of H_2_ formation on the TiO_2_(101) surface or subsurface as a function of the H/O ratio *σ* in the inner TiO_2_, respectively

During photocatalytic water splitting, H atoms are formed through the reaction of the photoexcited electrons with the protons that are produced in the proton coupled electron transfer steps of water oxidation^2^ (in agreement with previous studies of photocatalytic water splitting on anatase, O_2_ evolution is however not observed in our experiment, suggesting that the photoexcited holes are probably consumed to form free OH radicals^12,27–29^). Generally, there are three possible destinations for these H atoms^15^, namely: migration on the TiO_2_ surface, diffusion into the bulk TiO_2_, and desorption from the surface to form molecular H_2_. Previous computational studies reported that H_2_ recombinative desorption in vacuum environment is thermodynamically favorable but with a large energy barrier (~ 2 eV), making diffusion of hydrogen atoms into the bulk kinetically favored^13–15^. To explore the effect of the water environment on this process, we determined the energy barriers of surface-to-subsurface H diffusion on the clean surface and in the presence of adsorbed water by first-principles calculations. In agreement with previous studies^14,15^, we found that the diffusion process occurs via a surface three-fold oxygen (O_3c_) and can be divided into three steps (Fig. 2a, b). In step 1, the hydrogen atom, initially adsorbed at a bridging oxygen site, is transferred to O_3c_ and points away from the surface (state ‘sur3-1’); in step 2, H rotates around the O_3c_, so as to point toward the bulk (state ‘sur3-2’); finally, in step 3 H diffuses to a subsurface (sub) site. The potential energy profiles of H diffusion without and with adsorbed water are presented in Fig. 2b. We can see that the relative energies of the various intermediates as well as the energy barriers change significantly when the surface is covered with water molecules. In particular, the overall barrier is reduced from 1.13 to 0.77 eV, which makes the diffusion of hydrogen to the subsurface much easier in the presence of adsorbed water. These first-principle calculations thus support the idea that the water environment can effectively facilitate the diffusion of hydrogen to the subsurface of anatase, which ultimately will lead to the formation of a hydrogenated shell (Fig. 2c, d and Supplementary Fig. 4). This is consistent with our experimental finding of a hydrogenated surface shell around the anatase TiO_2_ NPs.

### Reactivity of the hydrogenated TiO_2_ shell

We next investigated the role of the hydrogenated shell in promoting H_2_ evolution by performing first-principle calculations of the energetics and kinetics of H_2_ formation on hydrogenated anatase (101) slabs^26,30–32^ with different H/O atomic ratios (*σ*) (Supplementary Movie 1). Our results are summarized in Fig. 2e, f, which show the energy barrier for H_2_ formation and desorption energies, respectively, both in the case where H_2_ is formed above and below the TiO_2_ surface (note that these results do not include entropic contributions, which tend to stabilize the H_2_ desorbed state at finite temperature), while the corresponding structures are shown in Supplementary Fig. 5. We can see that H_2_ formation has a prohibitive activation energy of over 2 eV on the non-hydrogenated surface, as already reported in previous studies^12^. However, the barrier is drastically reduced with increasing *σ*, becoming ~1 eV for H_2_ formation in the subsurface region of a hydrogenated slab with *σ* = 2/3. At the same time, the H_2_ diffusion barrier from the subsurface to the surface is obtained as only 0.24 eV. These results reveal that the critical role of the hydrogenated shell is to make hydrogen evolution kinetically viable by reducing the barrier of H_2_ (H–H bond) formation. Additional DFT results on the stability of the hydrogenated shell and its influence on water adsorption are reported in Supplementary Notes 3-5, Supplementary Figs. 6-9 (Supplementary Movie 2).

### Stability of the hydrogenated TiO_2_ shell

**Fig. 3:**
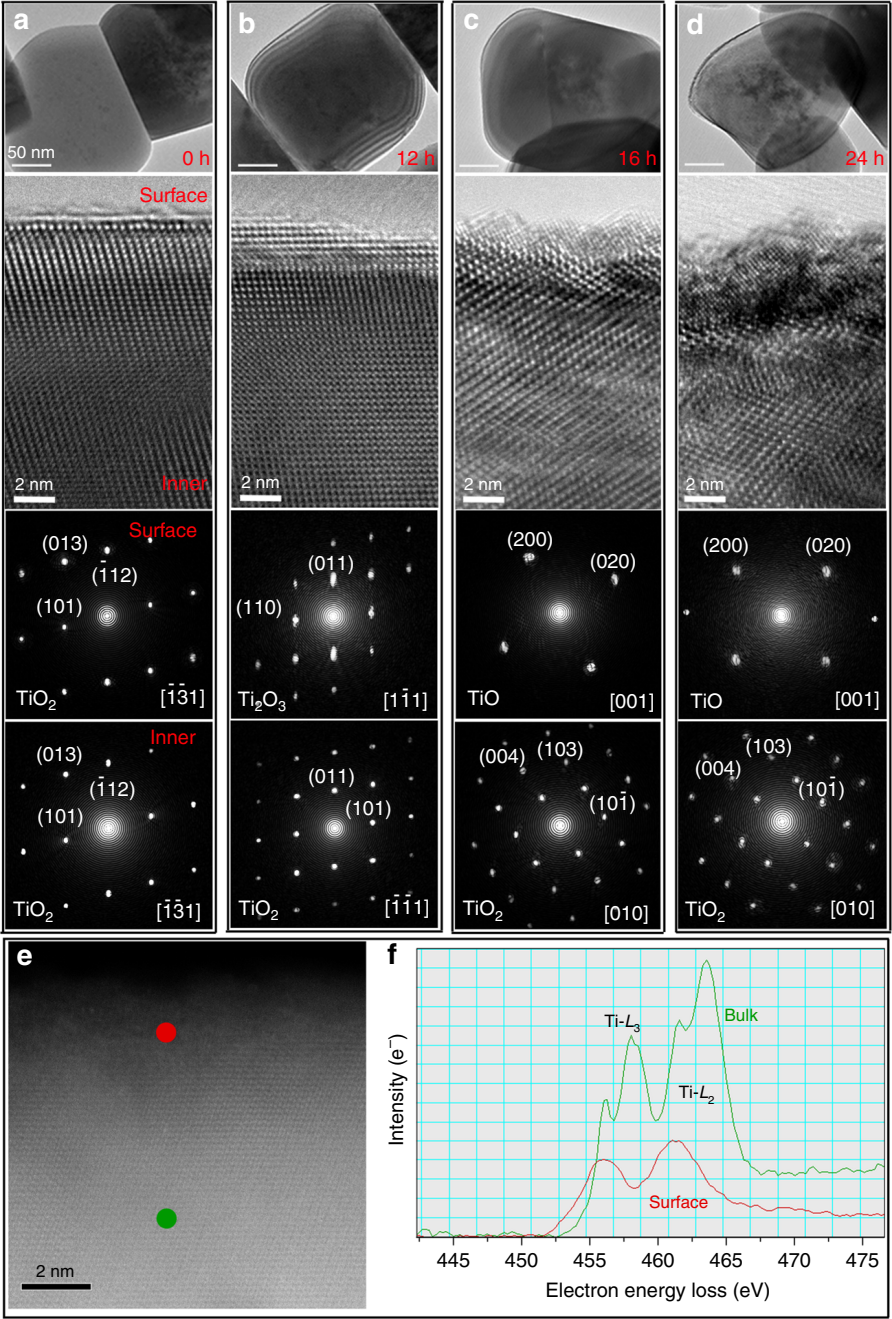
Ex situ observation of the recovery of the hydrogenated shell on TiO_2_ NPs. **a–d** Low magnification (top row) and high-resolution (second row) TEM images of dried TiO_2_ NPs after photocatalytic reaction with UV illumination in water for 0, 12, 16, and 24 h, respectively. Fourier transform (FT) patterns from the surface (third row) and internal bulk areas (fourth row) of the corresponding high-resolution TEM images in **a**–**d**, showing the TiO_2_, Ti_2_O_3_, and TiO structures, respectively. **e**, **f** HAADF image for the dried TiO_2_ NPs after photocatlytic reactions in water (for 16 h). The EELS were acquired from the surface (red dot in **e** and red spectrum in **f**), and internal bulk (green dot in **e**, and the green curve in **f**)

To further explore the stability of the hydrogenated shell on the TiO_2_ surface, we took the TiO_2_ NPs out from the aqueous solution after photocatalytic reaction and performed ex situ TEM analyses. High-resolution TEM (HRTEM) images in Fig. 3 show that the dried hydrogenated TiO_2_ shell transforms back to a crystalline structure with lower oxygen content and reduced Ti valence, evolving from Ti_2_O_3_ to TiO as the illumination time is prolonged. The EELS recorded from the surface shell changes in shape and onset position, shifting to lower energies in comparison to the EELS recorded in the bulk. This confirms that the Ti ions in the surface shell have a lower valence state relative to the Ti^4+^ state in the bulk (Fig. 3e, f). The thickness of the reduced crystalline shell on dried TiO_2_ NPs increases with the duration of UV illumination (Fig. 3b–d), which is consistent with the behavior of the hydrogenated shell in the wet state (Fig. 1c).

### Effect of Pt on the formation of hydrogenated TiO_2_ shell

**Fig. 4:**
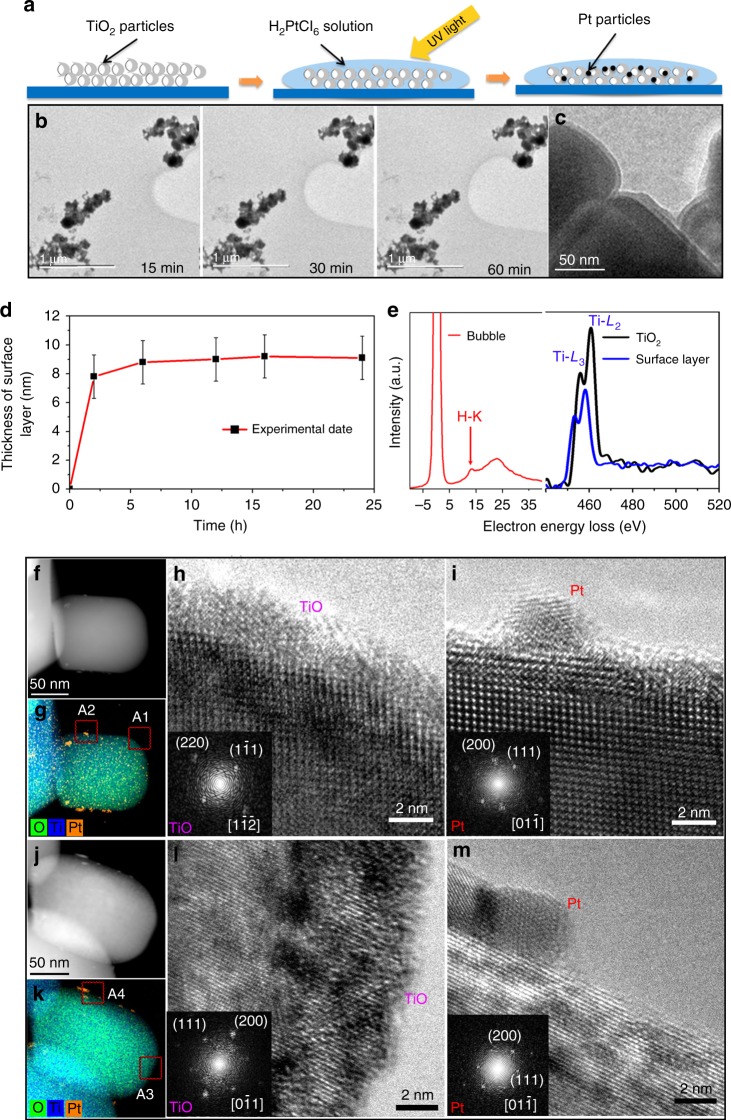
In situ LETEM observation of the photocatalytic water splitting process on TiO_2_/Pt and ex situ observation of the surface shell on TiO_2_/Pt NPs. **a** Schematic diagrams showing the deposition process of Pt on the TiO_2_ NP under UV light illumination for 4 h. **b** In situ LETEM observation of bubble evolution near the TiO_2_/Pt NPs in water under UV illumination for different period of times. The recording time of each TEM image was controlled within 1 s with an electron dose rate of about 3 e^−^ · Å^−2^ s^−1^. **c** A surface layer was observed on the TiO_2_/Pt NPs during the photocatalytic water splitting process. **d** Evolution of surface layer thickness on Pt- loaded TiO_2_ with increasing UV illumination time. **e** EELS of the bubble (red curve) at ~13 eV shows that the bubble contains hydrogen. Comparison between the EELS of pristine TiO_2_ (black curve) and surface layer on TiO_2_/Pt (blue curve) shows that the valence state of titanium in the surface layer has been reduced, same as the ones in Fig. 1f. **f** HAADF image and **g** EDS map of a TiO_2_/Pt NP, after UV photocatalytic reaction in water for 6 h. **h** HRTEM image of surface area far from Pt (A1 in **g**) and the corresponding FT pattern (inset) from the surface shell, which is identified as cubic TiO. **i** HRTEM image of surface area near a Pt nanodot (A2 in **g**) and the FT pattern for Pt nanodot (inset). **j** HAADF image and **k** EDS map of a TiO_2_/Pt NP after UV light illumination in water for 12 h. **l** HRTEM image of the TiO_2_/Pt NP in surface area far from the Pt co-catalyst (A3 in **k**) and the corresponding FT pattern identified as TiO (inset), showing that the structure of the surface shell on TiO_2_ is cubic TiO with thickness up to 4.5 nm. **m** HRTEM image of the TiO_2_/Pt NP in surface area near the Pt co-catalyst (A4 in **k**) and the FT pattern for Pt nanodot (inset)

The role of co-catalysts in photocatalytic water splitting on TiO_2_ has been intensively studied during the past few decades^11,12,33^. It has been recognized that the photoexcited electrons can be transferred from the conduction band to a Pt co-catalyst deposited on the surface of TiO_2_, which greatly reduces the possibility of electron–hole recombination and enhances the hydrogen evolution^11^. To explore the effect of Pt co-catalyst on the formation of the self-hydrogenated shell under UV illumination, we loaded 0.05 wt% Pt co-catalyst on the anatase TiO_2_ NPs surface by using H_2_PtCl_6_ solution under UV light illumination for 4 h, as schematically shown in Fig. 4a. After the loading of Pt, in situ observation of photocatalytic water splitting for The TiO_2_/Pt NPs was carried out into a LETEM. Fig. 4b shows bubble generation under UV illumination within 15 min and growth with increasing illumination time. The bubble was identified to be H_2_ by EELS with evidence of a peak at ~13 eV (red curve in Fig. 4e). Clearly, the loaded Pt on TiO_2_ surface effectively improves the efficiency of water dissociation and accelerates the hydrogen generation process, as shown in Supplementary Fig. 3b. At the same time, the hydrogenated TiO_2_ surface shell was also observed on the TiO_2_/Pt NPs (Fig. 4c). The thickening rate of surface shell with illuminating time (Fig. 4d) was much faster in the first couple of hours, as compared with that for the neat TiO_2_ NPs without co-catalyst. As in Fig. 1f, a comparison of the EELS spectra in Fig. 4e between the Ti–*L*_2_/*L*_3_ peaks for anatase TiO_2_ (black curve) and the surface shell (blue curve) indicates a reduction of valence state of titanium in the surface shell. The fast formation of the surface shell is mostly induced by the enhancement of hydrogen atom generation after Pt co-catalyst deposition, which inversely shortens the incubation time of hydrogen generation (Supplementary Fig. 3).

After illuminating the water-immersed TiO_2_/Pt using UV light for different lengths of time, the TiO_2_/Pt sample was dried out and the morphologic evolution of the surface shell on Pt loaded TiO_2_ NPs was further studied by ex situ TEM analysis. The high angle annular dark field (HAADF) images (Fig. 4f, j) and the corresponding energy dispersive spectroscopy (EDX) maps (Fig. 4g, k) clearly show that the co-catalyst Pt nano-dots are dispersed on the surface of TiO_2_ NPs. Two kinds of surface regions, with/without Pt loading, were observed in HRTEM images after about 6 h photocatalytic reaction. In the region (square A1 in Fig. 4g) far from the Pt nano-dots, the dried hydrogenated surface shell was noticeable with a thickness of ~2.5 nm (Fig. 4h) and the shell phase was identified as the cubic TiO structure based on the corresponding Fourier Transform (FT) pattern (inset in Fig. 4h). In contrast, no low-valence titanium oxide surface shell was observed in the region (square A2 in Fig. 4g) nearby the Pt nano-dots and the surface structure remains in the TiO_2_ phase (Fig. 4i). Again, for the TiO_2_/Pt sample after longer UV light illumination time of about 12 h, a TiO surface shell ~4.5 nm thick (Fig. 4l and inset) was observed in the area (square A3 in Fig. 4k) far from the loaded Pt nano-dots, whereas no low-valence titanium oxides were yet observed on the surface area nearby the Pt co-catalyst (A4, Fig. 4m).

All these results reveal that the hydrogenated TiO_2_ shell formed on the surface of the anatase TiO_2_ NPs both with and without Pt co-catalysts during photocatalytic reaction. The Pt co-catalysts enhanced both the formation of the hydrogenated TiO_2_ shell and the hydrogen evolution. However, the evolution of the hydrogenated TiO_2_ surface shell near the Pt co-catalyst is quite different from that of the areas far from the Pt nano-dots during the dehydration process. Instead of transforming to a low-valence titanium oxide layer as on the surface of TiO_2_ NPs without Pt loading (Fig. 3) or the local areas free of Pt nano-dots (Fig. 4h, l), the hydrogenated TiO_2_ surface shell nearby the Pt nano-dots just converted back to the original TiO_2_ structure by dehydrogenation (Fig. 4i, m). The main reason should be that H_2_ formation on Pt is easier than on TiO_2_, due to the smaller energy barrier of 0.85 eV^34^, which can effectively avoid H accumulation around the Pt.

We have developed a liquid environmental TEM for in situ visualization of the photocatalytic reaction at the interface between bulk water and anatase TiO_2_. We discovered the formation of a hydrogenated TiO_2_ surface shell many hours before the generation of hydrogen bubbles for anatase TiO_2_ without aid of co-catalyst Pt particles. The hydrogenated TiO_2_ shell was observed also on the surface of anatase TiO_2_ with Pt co-catalysts, along with hydrogen bubble generation. The shell thickness can be up to about 10 nm. This hydrogenated surface shell is formed via subsurface diffusion of H atoms generated by reaction of water protons with photoexcited electrons and promotes hydrogen generation by reducing the activation energy of H_2_ (H–H bond) formation. This discovery provides insight into the mechanism of hydrogen evolution on TiO_2_ that challenges the current understanding of photocatalytic surface chemical reactions without the aid of metal co-catalyst. It also enables a more effective exploration of novel highly efficient photocatalysts for water dissociation.

## Methods

### In situ TEM imaging for photocatalytic reaction on interface of water/TiO_2_ NP

A homemade liquid flow holder was used in this experiment for observation of the photocatalytic reactions in water, the tip of the liquid flow holder was assembled with the self-aligned wet (SAW) cells^35^ to seal the liquid. The SAW cell was covered by two 20 nm thick silicon nitride membranes, which is used to confine the liquid hermetically by O-ring for TEM observations. The gap between the two silicon nitride membranes can be controlled in a range from 5 nm to 1 µm for holding the liquid with a required thickness. A mercury light CEL-M500 (with characteristic UV wavelength of 254, 297, 315, 335, 365, 404, and 425 nm) was used in all the experiment. For the Pt co-catalyst TiO_2_, 0.1 g of TiO_2_ particles were suspended in water by sonication for one minute, then H_2_PtCl_6_ solution was directly injected to the solution for adjustment to 0.05 wt% Pt loading on the catalyst. After UV illumination the TiO_2_–H_2_PtCl_6_ solution for 4 h, the NP was centrifuged and dried at 313 K in vacuum. The TiO_2_ or Pt co-catalyst TiO_2_ aqueous suspensions with 0.1 mol L^–1^ is prepared by adding TiO_2_ NPs into the de-ionized water (DI water). The suspensions are injected into the liquid flow holder by a liquid pump outside. UV illumination is introduced into the LETEM by using an optical fiber, which can launch the UV light from the CEL-M500 mercury lamp with a wavelength range from 254 to 450 nm (100 mW cm^−2^ at the position of TEM observation area). A JEOL-2010F TEM equipped with a Gatan Enfina EELS system (energy resolution of 1.2 eV) is used at an operating voltage of 200 kV. The EDS mapping is operated on a Titan G2 60-300 Cs-corrected TEM with accelerating voltage of 300 kV, which is also equipped with a Gatan Dual EELS™ system.

### Theoretical models

First-principles calculations were carried out within the framework of density functional theory (DFT) as implemented in the CP2K/Quick step package^32^. CP2K/Quick step employs a hybrid Gaussian and plane wave basis set and norm conserving Goedecker-Teter-Hutter (GTH) pseudopotentials^36–38^. Gaussian functions consisting of a double-ζ plus polarization (DZP) basis set were employed to optimize the structures^37^. For the calculations of H_2_ formation above and below TiO_2_, the triple-ζ plus polarization (TZP) basis sets were employed. The energy cutoff for the real space grid used to represent the electron density was 500 Ry, which yields total energies converged to at least 0.001 eV per atom. We used the gradient-corrected Perdew–Burke–Ernzerhof exchange-correlation functional^39,40^ augmented with on-site Hubbard U term (equal to 4.2 eV) on the 3d orbitals of the Ti atoms and D3 dispersion corrections to account for van der Waals interactions^41–43^.

Our models typically consist of periodically repeated anatase (101) slabs with a (3 × 2) surface cell and three TiO_2_ tri-layers. *k*-space sampling was restricted to the Γ point. To avoid the interaction between adjacent slabs, a vacuum space of 15 Å was employed. Hydrogen diffusion was modeled using a (1×3) surface supercell with one extra hydrogen, corresponding to 1/6 ML coverage. Four adsorbed water molecules (2/3 ML) were added to study the effect of the water environment on hydrogen diffusion.

Born–Oppenheimer first-principles molecular dynamic (FPMD) simulations were performed within the canonical (NVT) ensemble at temperature of 300 K. The ionic equations of motion were integrated using the velocity Verlet algorithm with a time step of 0.5 fs. All the FPMD simulations were run for at least 10 ps.

### Data availability

The authors declare that the data supporting the findings of this study are available within the paper and its supplementary information. Further information is also available from the corresponding authors upon reasonable request.

## Electronic supplementary material

Supplementary Information

Description of Additional Supplementary Files

Supplementary Movie 1

Supplementary Movie 2
