# Supplementary material for: Self-hydrogenated shell promoting photocatalytic H2 evolution on anatase TiO2
Source: Nat Commun. 2018 Jul 16;9:2752. doi: 10.1038/s41467-018-05144-1 (PMC6048119; doi:10.1038/s41467-018-05144-1)
Supplement: Supplementary file 1 — Supplementary Information [file 41467_2018_5144_MOESM1_ESM.pdf]

Supplementary Information

Self-hydrogenated shell promoting photocatalytic H_2 evolution on anatase TiO_2

Lu et al.

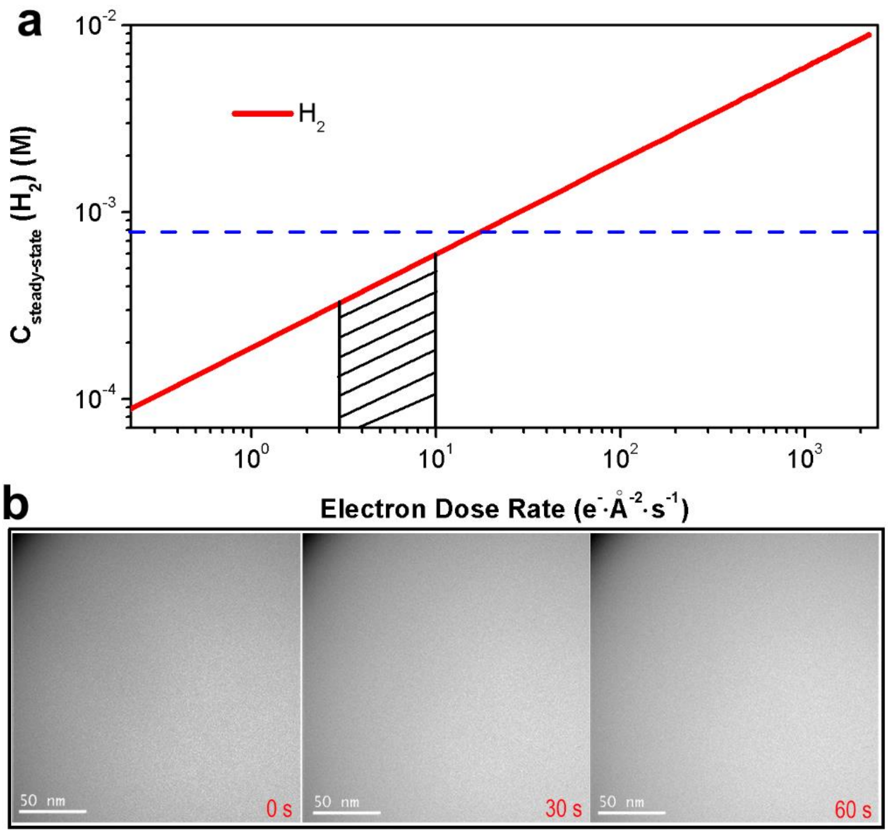

Supplementary Figure 1 | Hydrogen generation in radiolytic water. (a) The steady-state concentration of hydrogen $C_{steady\text{-}state}$ ($C_{ss}(H_2)$) in radiolytic water *vs.* the electron dose rate. Blue dash line indicates the saturation concentration of hydrogen in water at 100 kPa. The shadow area shows the experimental dose rates in this work and the corresponding steady-state concentrations are obvious lower than the saturation concentration. **(b)** *In situ* LETEM observations of the de-ionized (DI) water under the electron beam irradiation with a dose rate of ~1000 e$^-$·Å^{-2}·s^{-1}. No bubbles formed in the flowing DI water. (Supplementary note 1 has more discussion)

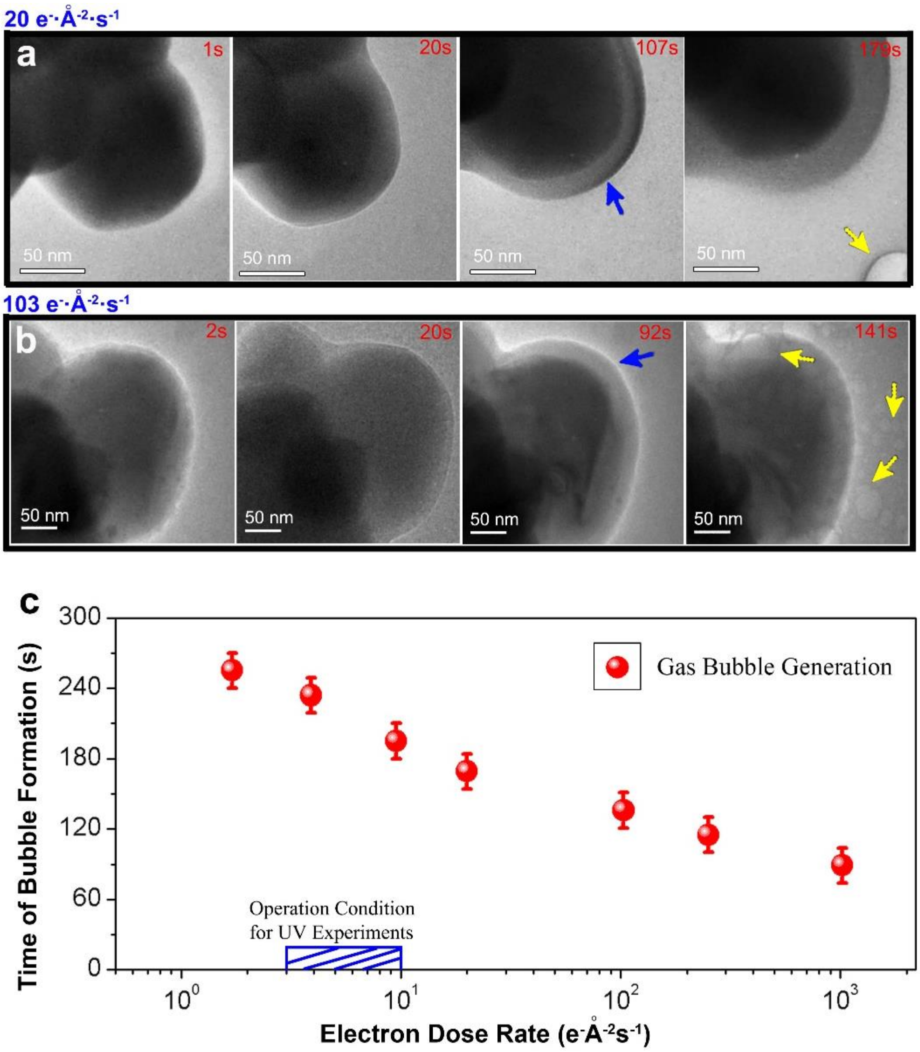

Supplementary Figure 2 | Bubble generation by electron beam irradiation in aqueous solution containing TiO_2 NPs without UV illumination. (a, b) *In situ* LETEM observations of the beam effect on aqueous solution containing TiO_2 NPs without UV illumination at a dose rate of 20 $e^{-}\cdot Å^{-2}\cdot s^{-1}$ and 103 $e^{-}\cdot Å^{-2}\cdot s^{-1}$, respectively. No obvious change is observed on both the TiO_2 NPs and the surrounding water before irradiation for 20 s. The blue arrows indicate the surface layer formed on the TiO_2 NPs and the yellow arrows show the bubbles formed in water. **(c)** Incubation time of bubble formation *vs*. electron dose rate. The blue shaded window shows the actual TEM operation condition of our UV phtocatalytic water splitting experiments, which ensures the bubble generation observed in this work is only from water splitting by UV illumination rather than as electron beam effect. (Supplementary note 2 has more discussion)

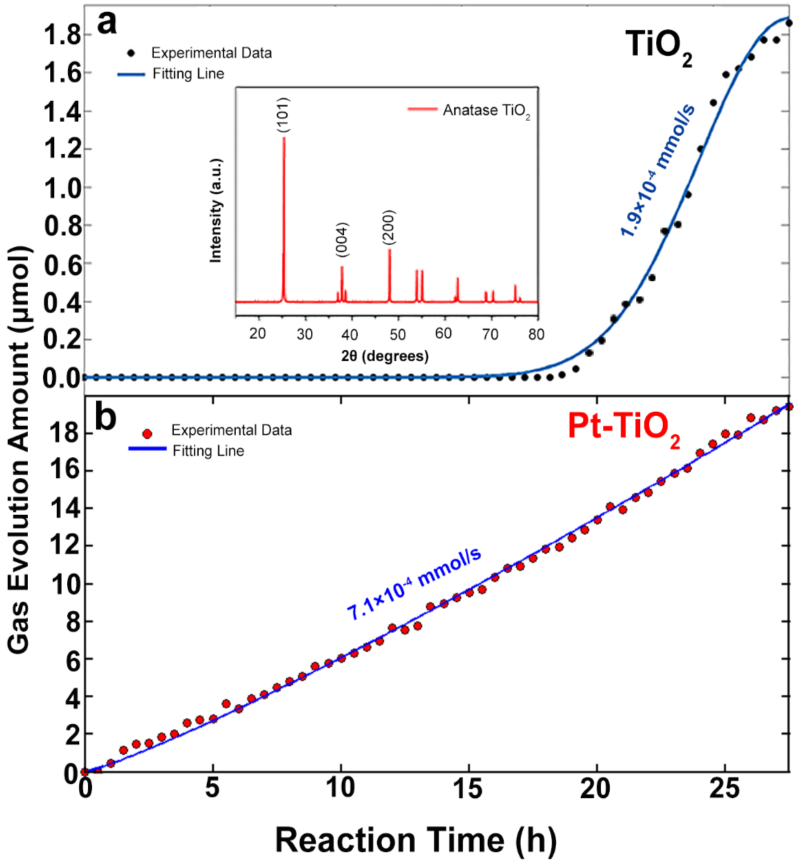

Supplementary Figure 3 | Kinetics of hydrogen bubble generation in aqueous solution of anatase TiO_2 with/without Pt co-catalyst during photocatalytic water splitting. (a) Amount of hydrogen generation in water containing TiO_2 NPs vs. UV illumination time (Anatase TiO_2, 20 mg; de-ionized H_2O, 50 mL). After an incubation time of ~18 h, the average gas evolution rate of hydrogen is about 1.9×10^{-4} mmol/s. Inset shows the XRD of the pure anatase TiO_2 NPs. **(b)** Gas evolution amount of hydrogen in TiO_2/Pt aqueous solution *vs.* UV light illumination time; from the fitted line, the average evolution rate of hydrogen is about 7.1×10^{-4} mmol/s.

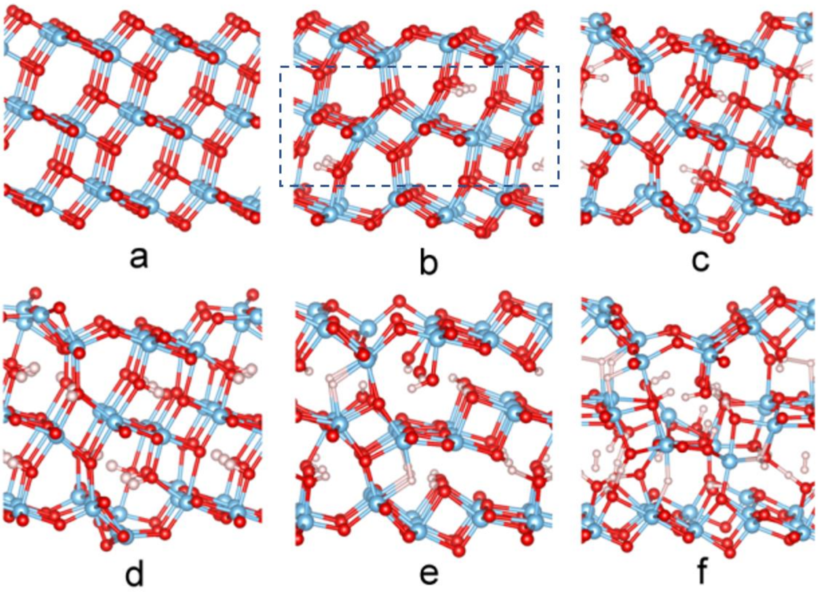

Supplementary Figure 4 | Atomic structures of the pristine and hydrogenated TiO_2(101) surface. **(a)** Stoichiometric anatase (101) slab containing three TiO_2 tri-layers. **(b-f)** Hydrogenated anatase (101) slabs with H/O ratio σ=1/6, 1/3, 1/2, 2/3, and 1, respectively. Red spheres are oxygen atoms, blue spheres are Ti atoms and white spheres are hydrogen atoms. σ is calculated as the ratio between the total number of H and O atoms beyond the surface layer, as is highlighted by the dashed line in **(b)**.

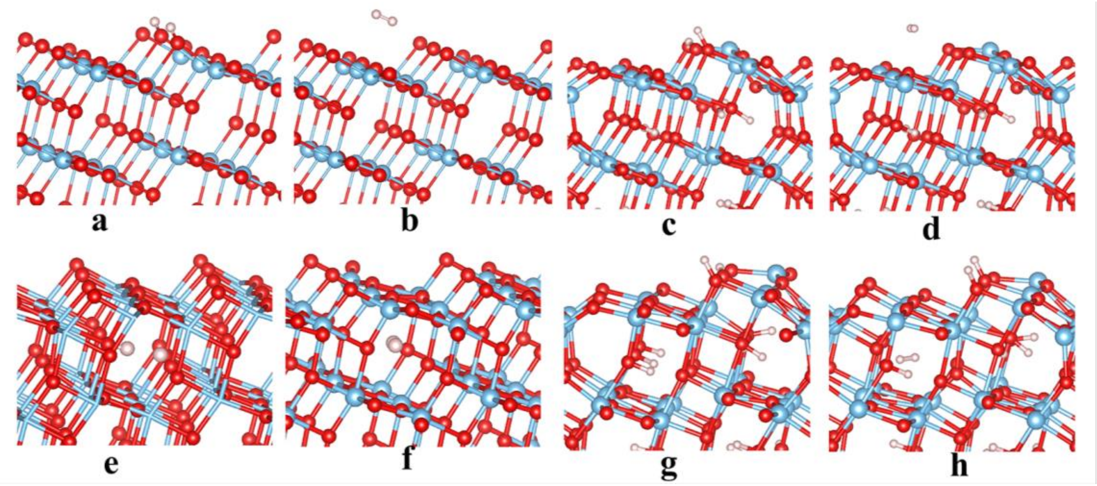

Supplementary Figure 5 | Atomic structures of the H_2 formation process from two adsorbed H atoms above and below anatase TiO_2(101). (a)-(d) show H_2 formation on the TiO_2(101) surface with σ = 0 and 1/3, **(e)-(h)** show H_2 formation in the subsurface with σ =0 and 1/3. **(a), (c), (e)** and **(g)** are initial states, and **(b), (d), (f)** and **(h)** are the corresponding final states of H_2 formation, respectively.

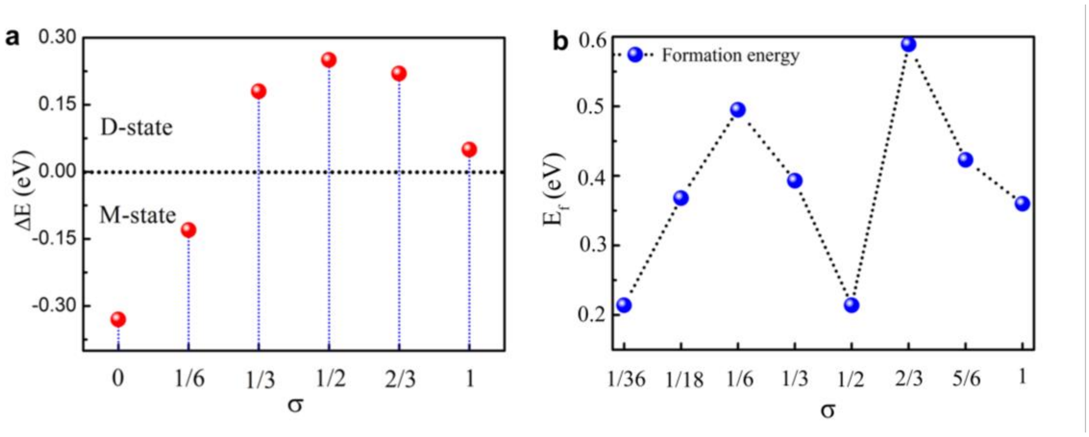

Supplementary Figure 6 | The dissociated (D-state)/molecular (M-state) state of water on hydrogenated TiO_2 and stability of hydrogenated TiO_2. (a) Computed energy difference (ΔE) between molecular and dissociated absorbed water on the hydrogenated TiO_2 surface as a function of the H/O ratio σ. Dissociation becomes favorable for $\sigma \geq 1/3$. **(b)** Formation energy E_f of the hydrogenated anatase (101) slabs as a function of the H/O ratio σ. (Also see Supplementary note 3)

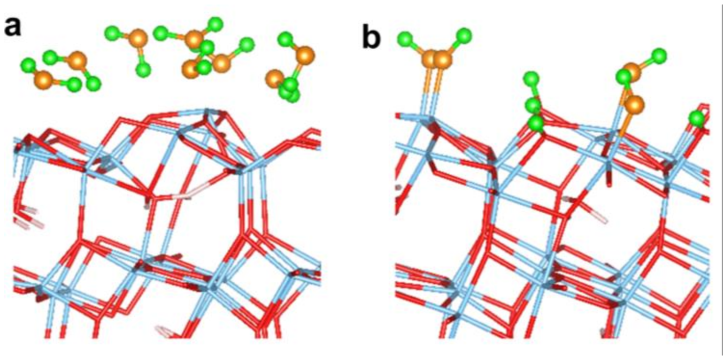

Supplementary Figure 7 | Water dissociation on hydrogenated anatase TiO_2(101) with σ=1/3 from FPMD. (a) Initial state of the simulation; **(b)** Configuration after 10 ps FPMD simulation of hydrogenated anatase TiO_2(101) in water at 300 K, showing four dissociated water molecules on the hydrogenated surface. For simplicity, only the water and TiO_2 layers at the interface are shown. The water O and H atoms are colored in orange and green, respectively. The Ti, O and H atoms of the hydrogenated slab are shown as blue, red and white sticks, respectively. (Also see Supplementary movie 1 and Supplementary note 3)

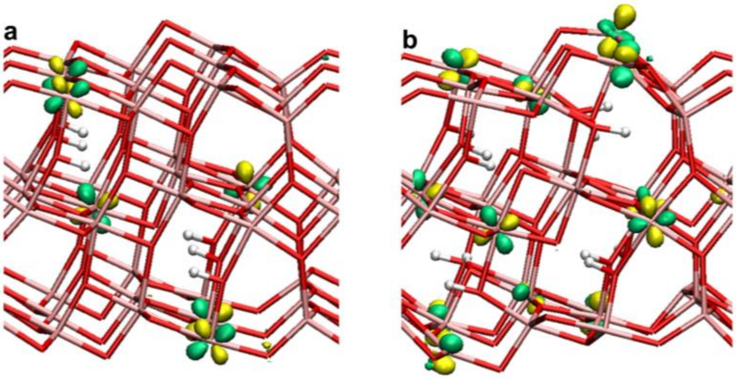

Supplementary Figure 8 | Spin density isosurface (10^{-2} a.u.$^{-3}$) of the hydrogenated anatase (101) surface. Excess electron distribution for the surface with (a) σ**=1/6**, and (b) σ**=1/3**. H atoms are represented by the white small spheres.

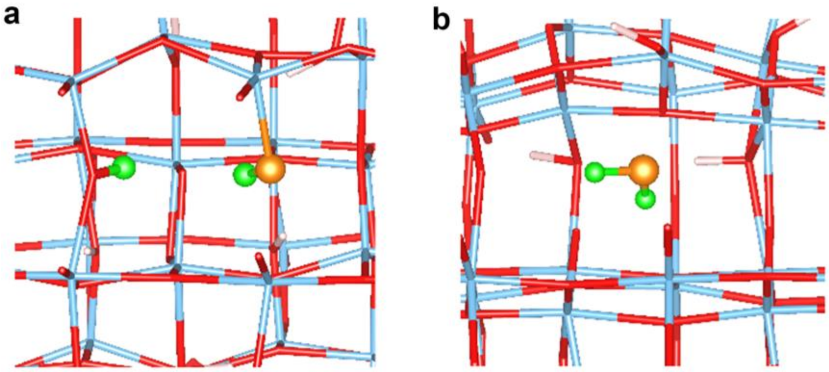

Supplementary Figure 9 | Stability of the hydrogenated TiO_2 slab with σ=1/3. Selected snapshots from a 10 ps FMPD simulation at 300 K: **(a)** Initial configuration with all the hydrogens bound to lattice oxygens; **(b)** Configuration after 2.5 ps where a water molecule is formed inside hydrogenated TiO_2 by proton transfer of one proton to one lattice oxygen that is already bound to another H; this results also in formation of an oxygen vacancy in the anatase slab. In order to highlight the water formation process, the water O and H atoms are shown in orange and green, respectively. The Ti, O and H atoms of the hydrogenated TiO_2(101) slab are shown as blue, red and white sticks, respectively. (Also see Supplementary movie 2 and Supplementary note 4)

Supplementary Note 1

Controlling the electron dose rate to reduce the beam effect on pure water

Under electron beam irradiation, electrons will transfer an amount of energy E to the atoms[1]. The maximum energy (E_{max}) an electron can transfer is

$$E_{max}=2E_0(E_0+2m_0c^2)/(Mc^2) \; ..... \; (1)$$

where m_0 is the rest mass of the electron, M is the mass of stationary nucleus, E_0 is the incident beam energy, c is the speed of light. Using the expression (1), we find that the maximum energy transferred to one water molecule is ~2.81 eV. Since the energy to split a water molecule according to $H_2O \rightarrow H_2 + ½ O_2$ is 2.46 eV (or 1.23 eV per electron)[2], an electron beam with E_0 = 200 keV can transfer enough energy for water splitting. It is reported that a water molecule can be decomposed into several radiolytic species[3] (such as solvated (or hydrated) electrons e_{aq}^{-}, hydrogen radical $H^{\bullet}$, hydroxyl radical ·OH, hydronium ion H_3O^+, hydroperoxyl radical $HO_2^{\bullet}$) as initially formed species and also some byproducts (such as hydrogen H_2, and hydrogen peroxide H_2O_2) as secondary species formed within a time in the order of few μsec[3,4]. However, the recombination rate of these radiolytic species is also very fast. For example, due to fast recombination rate of H_3O^+ and OH^- in water[5] (rate constant of ~1.4×10^{11} $M^{-1}s^{-1}$), the H_3O^+ and OH^- can recombine back to H_2O again in about ~20 ps[4]. As a result, a steady state concentration $C_{ss}(i)$ of the radiolytic species i will be established in water under electron beam irradiation. It has been reported that for the liquid cell electron microscopy the dependence of the steady state concentration on dose rate can be approximated with a power law [5]

$$C_{ss}(i) \sim \alpha_i \psi^{\beta_i} \text{ (M)} \quad (10^6<\psi<10^{10} \text{ Gy/s}) \quad (2)$$

For hydrogen molecules H_2 in radiolytic water, α_i is 8.88×10^{-8} and β_i is 0.5 in the case of homogeneous model, and ψ is the dose rate of electron beam with the unit of Gy/s. The dependence of steady-state concentration of hydrogen in radiolytic water on electron dose rate (with the unit of $e^-\cdot Å^{-2}\cdot s^{-1}$) is shown in Supplementary Fig. 1a.

To form a bubble from pure water, the dissolved gas concentration must significantly exceed the saturation concentration (C_{sat}) of hydrogen, which depends on the pressure of the system[6]. Previous reports have shown that the saturation concentration of hydrogen in water is ~0.77 mM at 100 kPa and ~3.15 mM at 400 kPa (reasonable value for the liquid cell) at room temperature, while the homogeneous nucleation needs a large supersaturation of ~190 mM[6].

During the photocatalytic water splitting experiments, the electron dose rate was controlled to be about 3-10 $e^{-}\cdot Å^{-2}\cdot s^{-1}$, which induced a steady-state concentration of hydrogen to be ~0.33-0.59 mM in water (see Supplementary Fig. 1a). These values are smaller than the saturation concentration of hydrogen in water even at a pressure of 100 kPa, so no bubble will generate during our experiments. It should be pointed out that no bubble was observed in water even when we increased the dose rate to ~1000 $e^{-}\cdot Å^{-2}\cdot s^{-1}$ (Supplementary Fig. 1b), corresponding to C_{ss} as high as ~5.95 mM[6]. The reason of without bubble formation may be attributed to the fact that the homogeneous nucleation needs a large supersaturation[6] in our system and the liquid flow may help to lower down of steady-state concentration of hydrogen in water.

Supplementary Note 2

Controlling the electron dose rate and recording time for TEM imaging to reduce the beam effect on aqueous solution containing TiO_2 NPs.

As discussed above, the irradiation of electron beam can induce water molecules to be decomposed into several radiolytic species[3] (such as hydrated electrons e_{aq}^{-}, hydrogen radical $H^{\bullet}$, hydroxyl radical $\cdot OH$, hydronium ion H_3O^+, hydroperoxyl radical $HO_2^{\bullet}$, hydrogen H_2, and hydrogen peroxide H_2O_2). Similarly, the energetic electron beam interacts with the metal oxides and desorption of oxygen atoms takes place via a core-hole Auger decay process[7,8]. Therefore, it is necessary to study the synergistic effect of electron beam on water and metal oxides.

The aqueous solution containing TiO_2 NPs was also studied by *in situ* LETEM in the absence of UV illumination. The morphological evolution of the TiO_2 NPs and the surrounding water were recorded as a function of irradiation time at different electron dose rates. As shown in Supplementary Figs. 2a and 2b, at the initial observation stage (a few seconds to less than 20 s), there was no visible change in both the TiO_2 NPs and the surrounding water. After that, a surface layer with visibly bright contrast grew gradually on the TiO_2 surface with increasing of irradiation time (as indicated by blue arrows in Supplementary Figs. 2a and 2b). After further prolongation of the irradiation time to about hundred and more seconds, gas bubbles started appearing in the surrounding water (as indicated by yellow arrows in Supplementary Figs. 2a and 2b). Evidently, the surface of TiO_2 NPs could be modified by electron beam effect but the bubbles formed in the aqueous solution containing TiO_2 NPs with an obvious incubation time. The incubation time of bubble formation is inversely proportional to the electron dose rate, as shown by a plot of the incubation time of bubble formation *vs.* the electron dose rate in Supplementary Fig. 2c. It is clearly shown that the bubbles formed after irradiation time longer

than a hundred seconds, as long as the electron dose rate was controlled less than 1000 $e^{-}\cdot Å^{-2}\cdot s^{-1}$.

In this work, we aim to investigate the photocatalytic reaction of anatase TiO_2 nanoparticles immersed in water by using LETEM with UV illumination. In order to reduce the beam effect on the aqueous solutions containing TiO_2 NPs during photocatalytic water splitting, each TEM image was recorded independently with fresh aqueous suspensions of TiO_2 NPs controlled by the fluidic holder, and the electron dose rate was controlled to be as low as about 3-10 $e^{-}\cdot Å^{-2}\cdot s^{-1}$ with about 1 second recording time for each frame of TEM imaging. A maximum operation time to get one frame of TEM image was controlled less than 20 seconds, which is ten times shorter than the bubble incubation time induced by the electron beam effect. The actual TEM operation conditions for the UV illumination experiments are located within the blue shadow area in Supplementary Fig. 2c. Such a TEM experimental design can perfectly avoid the formation of bubbles induced by electron beam effects in our photocatalytic water splitting experiment.

Supplementary Note 3

Water adsorption on hydrogenated anatase TiO_2

The energy difference between the molecular and dissociated state of adsorbed water depends on the H/O ratio σ, and the dissociated state becomes preferable with increasing σ (Supplementary Fig. 6a). In order to understand how the hydrogenated structure affects the water adsorption state, we carried out first-principle molecular dynamics (FPMD) simulations in water environment at the finite temperature of 300 K[9]. Hydrogenated slabs with σ=0, 1/6, 1/3, 1/2, 2/3, and 1 are shown in Supplementary Fig. 4. To study water dissociation by FPMD, we used a hydrogenated slab with σ=1/3 as a representative structure (see Supplementary Fig. 7 and Supplementary movie 1), and added 40 water molecules (with density 1 g/cm^3) into the region between two adjacent slabs to simulate the water environment. After about 1 ps, some water molecules began to adsorb on the anatase (101) surface with the O of water above Ti_{5c} and H pointing to the O_{2c} (Supplementary Figs. 4 and 6 and Supplementary Movie 1), in agreement with previous DFT results [10]. At about 2 ps, one water molecule started to dissociate by transferring one proton to a surface oxygen. Four water molecules were dissociated during our 10 ps FPMD simulation, see Supplementary Movie 1. On the other hand, no water dissociation was observed for the perfect anatase (101) in water during 10 ps FPMD simulation (using the same computational setting). These results confirm that the hydrogenated structure

substantially enhances water dissociation on the anatase (101) surface, consistent with the experimental results on hydrogen production.

Also, the first-principles calculations reproduce well the characteristics of reduced Ti^{3+} species. As shown in Supplementary Fig. 8, the excess electrons of hydrogenated TiO_2 are localized over the original Ti^{4+} ions, transforming them into Ti^{3+}. The amount of the Ti^{3+} species increases with the total amount of incorporated and adsorbed hydrogen.

Supplementary Note 4

Stability of the hydrogenated TiO_2

To determine the relatively stability of the hydrogenated slabs, the formation energy E_f has been calculated from the expression [11-13]:

$$E_f = \left(E_{slab} - E_{TiO_2} - nE_H\right)/n \quad (3)$$

Here E_{slab} is the total energy of the hydrogenated slabs, E_{TiO2} is the total energy of the perfect anatase TiO_2 slab, and E_H is the energy of the hydrogen atom. n is the number of hydrogen atoms. The results are shown in Supplementary Fig. 6b.

To investigate the stability of the hydrogenated layer, we focused on a TiO_2 slab with σ=1/3 and performed static calculations to check whether a water molecule can form inside the hydrogenated TiO_2 slab. We found that the water species is indeed stable inside the hydrogenated TiO_2 slab. The energy of the partially dehydrated TiO_2 slab with one water molecule relative to the hydrogenated TiO_2 slab is about -1.0 eV, which indicates that it is thermodynamically favorable for the hydrogenated TiO_2 to form water inside the material. To further verify this feature, a FMPD simulation was further carried out at 300 K. After 2.5 ps, one water is formed inside the hydrogenated TiO_2 slab, which is rather stable and does not convert back to the initial configuration, as shown in Supplementary Fig. 9 and Supplementary Movie 2, confirming that the hydrogenated TiO_2 is metastable.

Supplementary Note 5

xyz coordinates and cell parameters for relevant structures

The optimized atomic coordinates and cell information are listed. The unit is Angstrom.

1. The perfect and hydrogenated TiO_2 slabs with the different H/O ratio (σ)
    1. σ=0

2. σ=1/3
3. σ=2/3

2. Initial and final states of hydrogen diffusion process from the surface to the subsurface with or without water adsorption
    2.1. Initial state with H_2O on TiO_2
    2.2. Final state with H_2O on TiO_2
    2.3. Initial state without H_2O on TiO_2
    2.4. Final state without H_2O on TiO_2
3. H_2 formation on the hydrogenated TiO_2 with different H/O ratio (σ)
    3.1. Initial configuration for the isolated H atoms on TiO_2, σ=1/3
    3.2. Final configuration for H_2 on TiO_2, σ=1/3
    3.3. Initial configuration with the isolated H atoms inside TiO_2, σ=1/3
    3.4. Final configuration for H_2 on TiO_2, σ=1/3

Atomic coordinates:

1. σ=0

Lattice parameter: A 10.209915 B 11.328 C 25

Ti	6.1784887801	0.8726490284	5.8549561228
Ti	7.5934672352	0.8711281602	9.3445766901
Ti	9.0651075559	0.8695968420	12.9312932514
Ti	1.0713411286	2.7615210433	5.8546241100
Ti	2.4862598692	2.7615057542	9.3455444583
Ti	3.9603774989	2.7612841915	12.9338292708
Ti	3.8391003464	2.7613921382	6.4412444875
Ti	5.3138186517	2.7614563625	10.0295186478
Ti	6.7282701601	2.7614015377	13.5206507898
Ti	8.9443548741	0.8698392764	6.4438323709
Ti	0.2068534200	0.8710547191	10.0305205101
Ti	1.6210427432	0.8723445483	13.5203813181
O	4.2438789282	0.8740395075	6.4512993260
O	5.7155656288	0.8748097613	9.9963223719
O	7.2358560081	0.8707380606	13.7464844967
O	9.3503246956	2.7614632059	6.4532137226
O	0.6018507315	2.7615780236	9.9918829328
O	2.1284843213	2.7613270287	13.7484733594
O	2.0099736966	2.7613712573	7.3802262827
O	3.5518690555	2.7613807485	10.8944900605
O	5.1123208924	2.7611667660	14.3744397048
O	7.1169108493	0.8764149318	7.3824456315
O	8.6565312768	0.8604326336	10.8947962709
O	10.2167920265	0.8594480983	14.3737702696
O	0.5637465608	0.8709454722	5.6286977822
O	2.0847489158	0.8747546954	9.3787629853
O	3.5556376852	0.8737336545	12.9239415283
O	5.6708927193	2.7614750024	5.6265539919
O	7.1989895796	2.7615681538	9.3833677354
O	8.6591962019	2.7612880668	12.9218397224
O	2.6870603963	2.7613250369	5.0005310822
O	4.2481643623	2.7614965262	8.4805376867
O	5.7899756269	2.7614007067	11.9949409973
O	7.7925727603	0.8595415613	5.0014352645
O	9.3533897742	0.8606386563	8.4803618719
O	0.6829383591	0.8760054800	11.9927972898
Ti	6.1785408668	4.6508221139	5.8549208127
Ti	7.5932836224	4.6522171079	9.3447679258
Ti	9.0649905897	4.6528907525	12.9311290320
Ti	1.0735383704	6.5366541455	5.8549767314
Ti	2.4885042747	6.5351336223	9.3445807754
Ti	3.9601511163	6.5336056346	12.9312815797
Ti	3.8393949157	6.5338437876	6.4438206053
Ti	5.3118037675	6.5350575555	10.0305214620
Ti	6.7259996308	6.5363561532	13.5204007248
Ti	8.9445356973	4.6530354034	6.4437899148
Ti	0.2069227037	4.6523769127	10.0302241090
Ti	1.6209467748	4.6506825700	13.5203867848
O	4.2440191447	4.6492771592	6.4514742849
O	5.7160157186	4.6487029415	9.9965822556
O	7.2356699436	4.6518864392	13.7463131573
O	9.3488252900	6.5380413697	6.4512890231
O	0.6106007337	6.5388119698	9.9963264189
O	2.1309154178	6.5347459816	13.7465082002
O	2.0119648992	6.5404076311	7.3824551876
O	3.5515689792	6.5244267410	10.8947926527
O	5.1118237066	6.5234390077	14.3737577030
O	7.1167731426	4.6471578858	7.3824533050
O	8.6564582304	4.6622715585	10.8948770618
O	10.2166059740	4.6630847326	14.3736460714
O	0.5637461968	4.6522284849	5.6288221788
O	2.0843195441	4.6486992775	9.3783309421
O	3.5556714919	4.6489856711	12.9238124769
O	5.6687170767	6.5349478409	5.6287215789
O	7.1896991789	6.5387592551	9.3787678485
O	8.6605786114	6.5377457578	12.9239315637
O	2.6876104633	6.5235310332	5.0014171703
O	4.2484260433	6.5246312093	8.4803556960
O	5.7879075775	6.5399952686	11.9928005497
O	7.7927288298	4.6632530367	5.0014466411
O	9.3535252119	4.6625091087	8.4802375462
O	0.6831295639	4.6475763994	11.9927167817
Ti	6.1762875245	8.4255247792	5.8545947514
Ti	7.5912341833	8.4255081735	9.3455431418
Ti	9.0654058914	8.4252872942	12.9338594922
Ti	1.0736170863	10.3148217760	5.8549564116
Ti	2.4883323158	10.3162151941	9.3447844064
Ti	3.9600467487	10.3168819322	12.9311155917
Ti	3.8395497987	10.3170360633	6.4437744909
Ti	5.3118636978	10.3163762149	10.0302227880
Ti	6.7258915168	10.3146739173	13.5204045970
Ti	8.9440694007	8.4253950448	6.4412715950
Ti	0.2088544637	8.4254585076	10.0294946638
Ti	1.6232432330	8.4254035801	13.5206005801
O	4.2453742498	8.4254669907	6.4532196652
O	5.7068249596	8.4255799200	9.9918762549
O	7.2334864278	8.4253290365	13.7484331648
O	9.3489761465	10.3132789448	6.4514529366
O	0.6110589008	10.3127021774	9.9965821950
O	2.1307453277	10.3158781990	13.7463352268
O	2.0118164408	10.3111636920	7.3824878805
O	3.5514931780	10.3262739776	10.8948799168
O	5.1116263483	10.3270874358	14.3736432056
O	7.1149166506	8.4253687351	7.3802085585
O	8.6568269756	8.4253772994	10.8945139497
O	10.2172488790	8.4251647390	14.3745548466
O	0.5659191778	8.4254782416	5.6265175118
O	2.0940323999	8.4255705730	9.3833687672
O	3.5542135419	8.4252905616	12.9218695862
O	5.6686972044	10.3162286903	5.6288525125
O	7.1892586279	10.3126964361	9.3783375636
O	8.6606049815	10.3129761341	12.9238101036
O	2.6877823113	10.3272551278	5.0014003481
O	4.2485660623	10.3265127346	8.4802201112
O	5.7881067965	10.3115810540	11.9927090524
O	7.7920298402	8.4253194951	5.0005853906

O	9.3531288686	8.4254906257	8.4805370405
O	0.6850315980	8.4253968018	11.9948707128

2. σ=1/3

Lattice parameter: A 10.209915 B 11.328 C 25

Ti	6.0946405338	0.8674515106	5.8046882877
Ti	7.7082866144	0.8877540386	9.4122638738
Ti	8.6558517886	0.8590218017	12.9935193117
Ti	1.1301824008	2.7587354235	5.1374469520
Ti	2.5322845193	2.7565367570	9.2397013472
Ti	3.9665382546	2.7529940170	13.0196465227
Ti	3.8337431232	2.7524487943	6.2864468057
Ti	5.2687520012	2.7556413308	10.0664496671
Ti	6.6694343811	2.7585145024	14.1705346397
Ti	9.3532903635	0.8589732203	6.3111319263
Ti	0.0911495528	0.8884099911	9.8924394634
Ti	1.7048211271	0.8681158388	13.4999044465
O	4.1761394756	0.8964490580	6.4305285830
O	5.6093675420	0.8603695975	10.0784868413
O	7.1684649221	0.7705943104	14.2986138710
O	9.6396277247	2.7578995521	6.2534536925
O	0.5770798626	2.7568415952	9.7142521563
O	2.1410384751	2.7529454613	13.8279188341
O	1.9291089531	2.7595248453	7.1491645848
O	3.5162514746	2.7500989979	10.8649274395
O	4.8826326557	2.7605224468	14.5734147465
O	6.5258176455	0.8456253443	7.6310401189
O	8.7736201176	0.9078833556	11.1053094914
O	10.1659256978	0.8422056603	13.9781408312
O	0.6311886318	0.7713685170	5.0068697023
O	2.1901850709	0.8611029972	9.2266627666
O	3.6229990549	0.8971271843	12.8738434088
O	5.6582983686	2.7527101808	5.4774988842
O	7.2227081935	2.7564370659	9.5906500944
O	8.3685282770	2.7582647412	13.0519468736
O	2.9162984547	2.7606339094	4.7329813167
O	4.2838321359	2.7495455382	8.4402187594
O	5.8693349498	2.7592883475	12.1557410815
O	7.8431764876	0.8414267659	5.3258851811
O	9.2357584043	0.9079519843	8.1996349446
O	1.2736148580	0.8460382623	11.6737157033
Ti	6.0978794008	4.6291283358	5.8086065343
Ti	7.7077994460	4.6227684990	9.4146070059
Ti	8.6568562822	4.6570557152	12.9921621556
Ti	1.1981733849	6.5429076747	5.2718974273
Ti	2.5284252715	6.4909084757	9.2293407448
Ti	3.8162151087	6.5427618909	12.9743139184
Ti	3.9828847076	6.5420791222	6.3308351024
Ti	5.2719580677	6.4902359459	10.0765661613
Ti	6.6008973178	6.5430312536	14.0335957368
Ti	9.3527559713	4.6566835054	6.3134773084
Ti	0.0916474160	4.6231045528	9.8908181002
Ti	1.7016945204	4.6293795859	13.4968108644
O	4.1815895032	4.6041419532	6.4511898354
O	5.6075596199	4.6507941995	10.0800177602
O	7.1713535760	4.7448997984	14.2986960973
O	9.7074683269	6.5684563415	6.4531724005
O	0.5684250878	6.5429158217	9.6511644321
O	2.1249371591	6.4596704733	13.8945579267
O	1.9091478481	6.4472000844	7.1475772941
O	3.5054220696	6.5479412326	10.8694119659
O	4.8990369472	6.5268734715	14.5188396169
O	6.5280862772	4.6716705821	7.6341700168
O	8.7770433916	4.6077556655	11.1054365542
O	10.1648890654	4.6677621296	13.9786904992
O	0.6282848461	4.7441602149	5.0069705169
O	2.1924486585	4.6513450845	9.2258399474
O	3.6175844237	4.6044021055	12.8544880496
O	5.6742737818	6.4591704241	5.4105787955
O	7.2310952604	6.5425546388	9.6539202737
O	8.3014038554	6.5682465286	12.8516236005
O	2.9002181661	6.5266538259	4.7866075790
O	4.2943193277	6.5478295297	8.4363036274
O	5.8902236230	6.4472199366	12.1577456701
O	7.8445198380	4.6675433194	5.3270496532
O	9.2326382141	4.6079807447	8.2001038059
O	1.2711122509	4.6718148297	11.6713686987
Ti	5.9529692539	8.4130832399	5.8319531643
Ti	7.5672737307	8.4197390266	9.4734710812
Ti	8.7525185579	8.4217774599	12.9808934289
Ti	1.1987918574	10.2952504132	5.2717901238
Ti	2.5272831310	10.3506564039	9.2295453942
Ti	3.8150352167	10.2804953904	12.9734367774
Ti	3.9845007272	10.2798173720	6.3317292735
Ti	5.2715858173	10.3496738896	10.0748342030
Ti	6.6005390619	10.2951733191	14.0330490358
Ti	9.2560988522	8.4216676050	6.3224378717
Ti	0.2324536982	8.4199133425	9.8300800037
Ti	1.8463557227	8.4134295334	13.4718356530
O	3.9856807731	8.4118913563	6.4599250689
O	5.6782218644	8.4212998570	10.1424869956
O	7.2133400254	8.4209476902	14.2459995741
O	9.7086005193	10.2748835096	6.4536252308
O	0.5677885468	10.2951856144	9.6538718969
O	2.1222138043	10.3603726092	13.8934322804
O	1.9094868845	10.3992427599	7.1470828046
O	3.5059140908	10.2923384741	10.8702225454
O	4.8994754675	10.2749617237	14.5189339673
O	6.4732378593	8.4155056186	7.6796132220
O	8.8749272181	8.4210011569	10.9383467147
O	10.1962085308	8.4202211440	13.9384221908
O	0.5861262828	8.4208094157	5.0587437657
O	2.1214058729	8.4216061538	9.1623905088
O	3.8141177062	8.4125878790	12.8455723067
O	5.6771468743	10.3597701487	5.4113393896
O	7.2316173426	10.2944669533	9.6507474829
O	8.3006388588	10.2751122599	12.8514171809
O	2.8998368096	10.2745765480	4.7863751723
O	4.2934411047	10.2923913956	8.4344290894
O	5.8897316415	10.3992926128	12.1582680237
O	7.8125364158	8.4208837101	5.3639987282
O	9.1346284443	8.4212202815	8.3649537125
O	1.3273196020	8.4175576178	11.6238063719
H	2.1580298158	0.9113281643	11.2497476645
H	2.1565284853	4.6110226514	11.2490769843
H	2.2157385612	8.4152146943	11.2053336420
H	5.6412844277	0.9104866639	8.0547741253
H	5.6424863295	4.6103765370	8.0559770681
H	5.5847770351	8.4099655486	8.0980382188
H	6.8124532481	2.7521751792	11.8764174871
H	6.8227112643	6.4624211430	11.8170975220
H	6.8219997199	10.3806369014	11.8174082498
H	0.9876949345	2.7478537351	7.4337671980
H	0.9769930990	6.4620956473	7.4889366728
H	0.9767354544	10.3781473644	7.4866607528

3. σ=2/3

Lattice parameter: A 10.209915 B 11.328 C 25

Ti	6.2524627163	0.7621063314	5.6899234221
Ti	7.4578470988	0.7577325519	9.6755903888
Ti	8.4604449990	0.7973608980	13.0238721775
Ti	1.0936207039	2.6264481308	5.3225970857
Ti	2.7800635098	2.5446529304	9.1158277500
Ti	3.7816611774	2.6977398352	13.2829140076
Ti	3.9473776417	2.6688643970	5.9240247178
Ti	5.0934238702	2.5600595853	10.2390277576
Ti	6.4785847128	2.6712884333	14.3464289360
Ti	9.4876033756	0.7994427838	6.6180574250
Ti	0.4090865122	0.7437671576	9.5676174896
Ti	1.4691227958	0.8324699930	13.1893348017
O	4.2225776489	0.8147394084	6.2360734756
O	5.5622506700	0.7138858679	10.3300776737
O	7.0512950283	0.7836382560	14.4737576231
O	9.5750271476	2.6694888646	6.4278926832
O	0.6835711161	2.6092092354	9.4995955467
O	1.8919199772	2.7131376884	13.6714758941
O	2.2014621766	2.8122976328	7.0264414462
O	3.2759666631	2.8019796978	11.1393229068
O	4.7450037832	2.6999314759	14.8539070066
O	6.5462621204	0.3594686249	7.6071946458

O	8.5915468727	0.7498949775	11.0992693524
O	9.9596214324	0.8145481220	13.9365320245
O	0.6754452081	0.7217015624	5.1965248798
O	2.4320236879	0.6962604841	9.1602309785
O	3.4652692502	0.8219376863	13.1961738549
O	5.8097980270	2.6655871160	5.2832646867
O	7.1006275336	2.6070422113	9.6043393232
O	7.9690504124	2.6818343310	12.9536727702
O	2.8008472818	2.6031105693	4.5534005755
O	4.5866933719	2.7619429793	8.2967657206
O	5.6073706047	2.6151290290	12.4715098291
O	7.9866382607	0.7578491713	5.4231568055
O	8.9830458651	0.7530473461	8.5235553368
O	1.2281353492	0.7430980641	11.4819537314
Ti	6.2253828319	4.5655170169	5.6899345329
Ti	7.4468289220	4.5436231053	9.6895280614
Ti	8.4726207089	4.5636285337	13.0206597102
Ti	1.1232089732	6.4203100972	5.3292577188
Ti	2.7667755873	6.3221487867	9.1217792193
Ti	3.7813286018	6.4833290285	13.2801326755
Ti	3.9679438635	6.4631328949	5.9672043721
Ti	5.0732335169	6.3327712629	10.2417670746
Ti	6.4833373913	6.4381017272	14.3445720161
Ti	9.4826208658	4.5698186736	6.6145657934
Ti	0.3857605039	4.5115587298	9.5721440553
Ti	1.4822249213	4.6023738928	13.1920270047
O	4.2060533810	4.5531655730	6.1868691314
O	5.5618234421	4.4851675088	10.3736779206
O	7.0525738924	4.5543832626	14.4781811057
O	9.6353827500	6.4443007099	6.4463508790
O	0.6760500405	6.3786538642	9.5135281544
O	1.8887805649	6.4879418193	13.6693308438
O	2.1652510161	6.5846219614	7.0346426246
O	3.2662625053	6.5997891703	11.1407829571
O	4.7501727338	6.4856607775	14.8480528406
O	6.4981558498	4.2646457056	7.6240199131
O	8.6319009883	4.5400701825	11.0891322334
O	9.9684162330	4.5570245068	13.9305219611
O	0.7118844855	4.5583189474	5.2448299013
O	2.4306152087	4.4775496944	9.1507901031
O	3.4733321731	4.5979064258	13.1914644017
O	5.7834019298	6.4175727234	5.2476225622
O	7.0862969875	6.3751797129	9.6529663504
O	7.9945296399	6.4464375405	12.9896033655
O	2.7968331027	6.4200106107	4.5688914764
O	4.5589222804	6.4990329144	8.2704640493
O	5.6199495838	6.4156726202	12.4705922024
O	7.9680501780	4.5759980698	5.4428088890
O	8.9466214807	4.5204077723	8.5074688034
O	1.2536527442	4.4860026500	11.4822023060
Ti	6.1608883716	8.3440746081	5.6932678149
Ti	7.4398789136	8.2965507896	9.6814847739
Ti	8.4647779196	8.3471560935	13.0212584105
Ti	1.1358685197	10.2027931170	5.3237896742
Ti	2.7689119355	10.0935736099	9.1151350592
Ti	3.7717712547	10.2516243755	13.2802553776
Ti	3.9749593147	10.2284190564	5.9704698029
Ti	5.0861756527	10.1184062137	10.2340372048
Ti	6.4777174144	10.2243995658	14.3317228330
Ti	9.3990804134	8.3576205396	6.6063322836
Ti	0.3844735521	8.2848864310	9.5471153865
Ti	1.4709583915	8.3725687620	13.1911656097
O	4.1546061975	8.3513694553	6.2217276214
O	5.5358030466	8.2658116505	10.3420749536
O	7.0416230652	8.3382314100	14.4630942855
O	9.6579785785	10.2123951852	6.5103697059
O	0.6744494483	10.1514012096	9.5021544905
O	1.8870535058	10.2660434555	13.6654291917
O	2.1856845784	10.3952403858	7.0196413118
O	3.2765408594	10.3677447319	11.1456680479
O	4.7475698181	10.2491168773	14.8435888909
O	6.4919193024	7.9251278482	7.6350772266
O	8.6031827741	8.2830548840	11.1014965461
O	9.9642510512	8.3573206238	13.9337050174
O	0.6742924967	8.3230128964	5.2526837394
O	2.4340804227	8.2567227598	9.1389434317
O	3.4709095253	8.3688081424	13.1949365605
O	5.7884407874	10.2582126107	5.2287337312
O	7.1020301722	10.1447198665	9.6432311752
O	7.9849820471	10.2292904539	12.9673153474
O	2.8074399124	10.1682699380	4.5654815936
O	4.5753291769	10.2812922784	8.2656632091
O	5.6117031713	10.2113069487	12.4625957014
O	7.9669519823	8.3733667394	5.4868057717
O	8.9342812092	8.2858717869	8.5125762331
O	1.2588768940	8.2715633874	11.4804364975
H	0.2134520571	2.6004974071	11.8045735949
H	0.2409956122	6.3902699678	11.7711644422
H	0.2305565019	10.1753639059	11.7963136322
H	6.9997898504	-0.5079690656	7.6383573615
H	7.0770443855	3.4558725747	7.6192915729
H	6.9304034773	7.0477025903	7.6240103386
H	6.5554597121	2.6964625923	12.1651295374
H	6.5581609948	6.4916465607	12.1482274948
H	6.5560190021	10.2628977210	12.1462491355
H	1.9248712633	3.7339172998	7.2866297799
H	1.8068646986	7.4703396914	7.3249067531
H	1.8383487049	11.2932560484	7.3034205055
H	9.7004178548	2.6552908640	11.8642651430
H	9.7434861399	6.4220037044	11.9280109488
H	9.7267668900	10.1912951511	11.9005176141
H	10.9713617648	0.8069208697	7.7550105965
H	10.9386024018	4.5477161576	7.7882920475
H	10.9278867490	8.3088433512	7.7949263873
H	2.6681747216	3.6056198502	11.2069284688
H	2.6567758142	7.4010354347	11.2062009131
H	2.6962812553	11.1781144584	11.1966190062
H	5.1680888192	3.5425362459	8.0237509455
H	5.2098372866	7.2668674160	8.0378561824
H	5.2103942147	11.0381754827	8.0271582080

2.1 Initial state with H_2O on TiO_2

Lattice parameter: A 10.209915 B 11.328 C 25

Ti	6.1825403308	0.8309035861	7.4506029022
Ti	7.5922077102	0.8347082483	10.9402041895
Ti	9.0454545328	0.8358750008	14.5134604880
Ti	1.0781049765	2.7107323959	7.4562076034
Ti	2.4876217495	2.7159974894	10.9467424044
Ti	3.9300781234	2.7117639734	14.5162807017
Ti	3.8417383155	2.7116289061	8.0663715601
Ti	5.3140681343	2.7160067079	11.7051782633
Ti	6.6933715327	2.6964918239	15.2837052851
Ti	8.9460184711	0.8303689946	8.0600549737
Ti	0.2087309848	0.8338425287	11.6893927504
Ti	1.6028328284	0.8419854060	15.2749092406
O	4.2453125298	0.8271872676	8.0539098235
O	5.7016753912	0.8268873587	11.6106115162
O	7.1851695806	0.7947629138	15.3963067680
O	9.3505503266	2.7116980862	8.0509953180
O	0.5904652627	2.7087341379	11.6039094644
O	2.1059950092	2.7053430683	15.4268092292
O	2.0121725308	2.7114259897	8.9869822714
O	3.5316282638	2.7608040830	12.5080530982
O	5.0253009307	2.6826103966	16.0449872928
O	7.1170850991	0.8254298228	8.9803719333
O	8.6442077808	0.7868199661	12.4953857706
O	10.1326904696	0.8582151988	16.0330684608
O	0.5644673813	0.8279020566	7.2259194389
O	2.0864811462	0.8280736651	11.0017947252
O	3.4776391000	0.8327567376	14.4369760561
O	5.6664493398	2.7099698998	7.2237705634
O	7.1666271780	2.7086052452	10.9775229543
O	8.5695218602	2.6869340718	14.4385276437
O	2.6923195742	2.7026774670	6.6100370032
O	4.2427640741	2.7130499981	10.1131993487
O	5.7438447647	2.6768266192	13.6727075946
O	7.7966409261	0.8395563471	6.6043466687
O	9.3506679721	0.8339761861	10.1011160732
O	0.6422759051	0.8498811979	13.6709858865
Ti	6.1779421126	4.5995875365	7.4264440179
Ti	7.5725309885	4.5985816541	10.8992745941
Ti	8.9853662370	4.5612541999	14.4334919710
Ti	1.0702016991	6.4899767525	7.4378655480
Ti	2.4492943363	6.4772172708	10.9011836436
Ti	3.8298064479	6.4637647758	14.3803921854

Ti	3.8449862723	6.4913332806	8.0486301431
Ti	5.3202184996	6.4958408415	11.6690135314
Ti	6.8133960950	6.5127536749	15.1799125365
Ti	8.9479570199	4.6003831308	8.0575059568
Ti	0.1897449924	4.5954783276	11.6895664831
Ti	1.5531235407	4.5844741398	15.2613714543
O	4.2440980786	4.6032665105	8.0531435141
O	5.7044699819	4.6108239710	11.6479347792
O	7.0852961202	4.6171802846	15.2648857250
O	9.3508051569	6.4915547227	8.0459482117
O	0.5840139129	6.4925140556	11.5987787389
O	2.0694084179	6.4913141220	15.3405855363
O	2.0059583043	6.4904924467	8.9723556645
O	3.4913699362	6.4477320647	12.4967137113
O	4.9732468572	6.5156823621	16.1598587070
O	7.0941469009	4.6032453391	8.9605845837
O	8.5835604090	4.6008394288	12.4338836956
O	9.8619854865	4.6117615011	16.0326655112
O	0.5657692651	4.6020509630	7.2241615848
O	2.0553778718	4.6017183685	10.9749699413
O	3.5673866090	4.6091629412	14.5771153402
O	5.6644665828	6.4934012929	7.2170449770
O	7.1596296328	6.5017932330	10.9774413399
O	8.5165936845	6.5205509574	14.3790056231
O	2.6917377905	6.5031621202	6.6004030975
O	4.2360440824	6.4912774335	10.1102904220
O	5.6837686579	6.5646963789	13.6958954849
O	7.7968322961	4.6014326198	6.5954451859
O	9.3347033415	4.6021955964	10.0686237659
O	0.5230430686	4.5925086940	13.6127754390
Ti	6.1796432089	8.3752973834	7.4415242122
Ti	7.5859319575	8.3845898895	10.9213799033
Ti	9.0279122293	8.4296510030	14.4693443680
Ti	1.0683200156	10.2677228151	7.4409469172
Ti	2.4612188642	10.2675595861	10.9129735833
Ti	3.8547000453	10.2650173664	14.4246754349
Ti	3.8445210318	10.2670352956	8.0368592195
Ti	5.3101746543	10.2711644723	11.6079319148
Ti	6.6342855240	10.2877942039	15.1330447604
Ti	8.9469316864	8.3739214649	8.0512433058
Ti	0.2089693209	8.3751783296	11.6696744042
Ti	1.5745511573	8.3589419720	15.2598496593
O	4.2448020553	8.3751168339	8.0522365541
O	5.6984912777	8.3799123261	11.6029517629
O	7.1595833590	8.4237059560	15.3565457693
O	9.3513961965	10.2653854108	8.0511615994
O	0.6122712412	10.2668444293	11.6073086385
O	2.0584495941	10.2607781203	15.2851055991
O	2.0006007895	10.2674175191	8.9710811920
O	3.5341812605	10.2530807811	12.4560442865
O	4.9725273555	10.3307612281	15.9669704201
O	7.1115240235	8.3745599209	8.9725645490
O	8.6370445041	8.4225963259	12.4789213024
O	10.0969633414	8.3336773035	15.9970438553
O	0.5629727747	8.3746534998	7.2195088133
O	2.0705353545	8.3693354740	10.9770964370
O	3.4942054466	8.3655904251	14.4735450753
O	5.6696201793	10.2659185636	7.2210584860
O	7.1701738357	10.2678242416	10.9617075492
O	8.5983436123	10.2751182216	14.5223724250
O	2.6878774165	10.2658951082	6.5951331990
O	4.2429201617	10.2670317032	10.0637713625
O	5.7352282447	10.2671206332	13.5912875486
O	7.7955330112	8.3644346753	6.5984268410
O	9.3478061866	8.3695786681	10.0862694677
O	0.6331067801	8.3765330091	13.6439033865
O	8.0111118708	6.4348660691	17.0990760658
H	8.7334307386	5.7694859436	16.9024154444
H	8.5121358766	7.2740706070	17.1662747589
H	4.8624318626	5.6960570759	16.6727405606
O	8.0062122100	2.8251839781	17.1558619864
H	8.4876139421	1.9777903845	17.2396656696
H	8.7362164668	3.4668746910	16.9191969188
O	2.7604382443	8.3490097069	17.2235873252
H	3.5820536650	7.8385447558	17.0330202866
H	3.0760608510	9.2496585203	17.4208650805
O	2.9726110290	0.7015328934	17.2106741408
H	3.7594233135	0.1778135896	16.9077118882
H	3.3564373206	1.5926982978	17.3276852573

2.2 Final state with H_2O on TiO_2

Lattice parameter: A 10.209915 B 11.328 C 25

Ti	6.1802960586	0.8326730766	7.4390438351
Ti	7.5997174094	0.8000481614	10.9245107129
Ti	9.0117474854	0.8137253526	14.5146282640
Ti	1.0610857013	2.7216826126	7.4062956463
Ti	2.3991355619	2.7079979590	10.8240564963
Ti	3.9033952818	2.6829063538	14.6888583881
Ti	3.8533242201	2.7214182208	8.0560139212
Ti	5.3818931005	2.7063061236	11.7018008388
Ti	6.7123863077	2.7000118118	15.2573838446
Ti	8.9435140780	0.8373722210	8.0640003559
Ti	0.2017914499	0.8342142867	11.7292787065
Ti	1.5825161307	0.8351431156	15.2885107154
O	4.2421690207	0.8388304110	8.0497734513
O	5.6825973425	0.8285669640	11.6071226304
O	7.1681603293	0.7872306580	15.3600072726
O	9.3514995624	2.7194053183	8.0468557862
O	0.5546186097	2.7114495262	11.5624844178
O	2.0979044919	2.6517637786	15.5082969018
O	1.9869825619	2.6972396223	8.9590753276
O	3.4138941043	3.0304961378	12.5681667329
O	5.0441140579	2.6203346930	16.1012930951
O	7.1045058006	0.8429958488	8.9734814960
O	8.6319906335	0.7844521695	12.4889895530
O	10.0976555227	0.8498272668	16.0261227811
O	0.5617471299	0.8398925610	7.2114073392
O	2.1159618372	0.8522357567	11.0545533549
O	3.4658691886	0.8474436801	14.3819552475
O	5.6587383710	2.7192516277	7.2169357840
O	7.1537964262	2.7068930768	10.9917823048
O	8.5268868110	2.6895782970	14.4161972874
O	2.6965169936	2.7420484140	6.6030933630
O	4.2040894049	2.6886460149	10.1534987678
O	5.6717781764	2.6841969006	13.6774581493
O	7.7964636974	0.8434944065	6.6020577032
O	9.3377337644	0.8274888433	10.0989720322
O	0.6290043609	0.8582299582	13.6659557768
Ti	6.1770626832	4.6111046150	7.4223234439
Ti	7.5696406428	4.6260213439	10.8926690726
Ti	8.9365313553	4.6109226472	14.4316515576
Ti	1.0722714818	6.5044566353	7.4401884453
Ti	2.4682370952	6.4915814022	10.9277862617
Ti	3.8772580658	6.4967752492	14.5297437137
Ti	3.8377073136	6.5015432765	8.0664261541
Ti	5.2982524366	6.4938326125	11.7141435415
Ti	6.6598677580	6.5118872988	15.2746962580
Ti	8.9450310800	4.6106536170	8.0635092308
Ti	0.1776638096	4.6145063400	11.7154094564
Ti	1.5097850239	4.5923634925	15.2813717415
O	4.2426686320	4.6043381847	8.0654238895
O	5.6817920848	4.6018213370	11.6055942662
O	7.0903456704	4.6017003606	15.2763716238
O	9.3453075756	6.4998876753	8.0391530086
O	0.5705510099	6.5027684373	11.5935209647
O	2.0649191572	6.5329903783	15.3406863800
O	1.9959152472	6.5073024007	8.9738756313
O	3.4972338006	6.3789249388	12.5019196287
O	4.9767250630	6.5920800268	16.0371679551
O	7.0891437190	4.6009031567	8.9589423215
O	8.5752404162	4.6035418152	12.4417213836
O	9.9050550891	4.5841654651	16.0088102881
O	0.5628564905	4.6055096713	7.2205755693
O	2.0353938781	4.6043163479	10.9632797768
O	3.4834161464	4.5913844746	14.6407556375
O	5.6627636022	6.4973034376	7.2185570451
O	7.1591961018	6.4964539689	10.9791814232
O	8.5240624874	6.5131657941	14.4322057008
O	2.6915846546	6.5035560562	6.6046687879
O	4.2216856051	6.5035010277	10.1075449781
O	5.7104268775	6.5077689021	13.6605631596
O	7.7958094663	4.6038232983	6.5959343059
O	9.3124801529	4.6123960639	10.0697713798
O	0.5299777621	4.5951783699	13.6090889466

Ti	6.1799820670	8.3788826515	7.4367221408
Ti	7.5876936393	8.3678469951	10.9246940018
Ti	9.0024557567	8.3735593406	14.5003928025
Ti	1.0680409260	10.2735014653	7.4404386104
Ti	2.4594368224	10.2611641566	10.9145184315
Ti	3.8400373206	10.2636616548	14.4207448725
Ti	3.8439748561	10.2708710615	8.0412895160
Ti	5.3026149379	10.2569793501	11.6110928515
Ti	6.6307760037	10.2691910773	15.1156262654
Ti	8.9428039931	8.3871701925	8.0630066986
Ti	0.1993795678	8.3775274818	11.7054011176
Ti	1.5697277949	8.3539078331	15.2619479948
O	4.2387611396	8.3833192514	8.0458269922
O	5.6851239448	8.3727278277	11.5946436363
O	7.1501591248	8.4212100415	15.3664065070
O	9.3506324505	10.2771686964	8.0524989768
O	0.6046469036	10.2792938966	11.5922608960
O	2.0612242466	10.2673243197	15.2799804437
O	1.9967372000	10.2869287626	8.9751890577
O	3.5332145117	10.2626121213	12.4426310765
O	4.9631855215	10.3353625053	15.9533361803
O	7.1028319328	8.3652731265	8.9692413440
O	8.6187571798	8.4080923449	12.4795344545
O	10.0907893594	8.3324668563	16.0061037483
O	0.5621566992	8.3899576905	7.2246484081

O	2.0806108800	8.3786510510	11.0124621950
O	3.4816301934	8.3849062529	14.4249458325
O	5.6664027876	10.2731495053	7.2224477582
O	7.1660017148	10.2654111071	10.9574647143
O	8.5831115970	10.2655697446	14.5143959761
O	2.6859257048	10.2690464463	6.5967318644
O	4.2373828347	10.2670767803	10.0568307826
O	5.7292695271	10.2544779246	13.5720568102
O	7.7957197745	8.4075454492	6.6003300611
O	9.3361256075	8.3827826993	10.0907371638
O	0.6106780174	8.3830457258	13.6425008451
O	7.9479950776	6.4430037013	17.1589939761
H	8.6605116940	5.7804717869	16.9673596345
H	8.4532430737	7.2809762036	17.2039250746
H	3.3157578995	4.0014708088	12.7058429358
O	8.0105921723	2.7632775857	17.1524624921
H	8.4994625264	1.9164805854	17.2060656081
H	8.7353741336	3.4088366259	16.9329003877
O	2.7635800668	8.3577915852	17.2223948818
H	3.5507381888	7.7814765509	17.0812621944
H	3.1400588667	9.2430180856	17.3817676020
O	2.9709708727	0.6267914487	17.2148859646
H	3.7835131086	0.1480122164	16.8999438412
H	3.3155558446	1.5109180063	17.4411760899

2.3 Initial state without H_2O on TiO_2

Lattice parameter: A 10.209915 B 11.328 C 25

Ti	6.1714604737	0.8737505576	7.4342026789
Ti	7.5986058053	0.8709517664	10.9143890528
Ti	9.1243027325	0.8621748460	14.5081287260
Ti	1.0728211053	2.7633765196	7.4526544311
Ti	2.4949396654	2.7569585815	10.9425453460
Ti	3.9711847482	2.7389747107	14.5303884429
Ti	3.8392125469	2.7644582292	8.0385818688
Ti	5.3211966685	2.7611333304	11.6170818099
Ti	6.7539456995	2.7284261964	15.0745789331
Ti	8.9379790258	0.8752541127	8.0139620360
Ti	0.2134186854	0.8648440000	11.5970961609
Ti	1.6768786474	0.8479796235	15.0805996998
O	4.2392878334	0.8755227752	8.0404473953
O	5.7181925852	0.8692228425	11.5627638147
O	7.2919408194	0.8502779315	15.3418120006
O	9.3445613993	2.7631586241	8.0338015212
O	0.6066408378	2.7476695084	11.5353894124
O	2.1911085856	2.6713071395	15.3495167456
O	2.0125849992	2.7631143198	8.9760058158
O	3.5550613462	2.7687970448	12.4983356767
O	5.1634171884	2.7582556028	15.9642905152
O	7.1127367876	0.8572142111	8.9553310885
O	8.6719020657	0.8771078945	12.4582600625
O	10.2696000333	0.8400268715	15.9447013996
O	0.5667017634	0.8761300932	7.2195949332
O	2.1161397421	0.8660602945	10.9768785019
O	3.6208970162	0.8401946774	14.5054362102
O	5.6671026261	2.7640708821	7.2199779167
O	7.1767394602	2.7571509968	10.9188552482
O	8.7002028312	2.7299686416	14.5075384153
O	2.6867968369	2.7642203226	6.5960442221
O	4.2491173421	2.7635947285	10.0730022845
O	5.8072784521	2.6969542662	13.5611390162
O	7.7800835801	0.8854052553	6.5732902294
O	9.3526592013	0.8573857082	10.0468088281
O	0.7128642162	0.8543259136	13.5522504893
Ti	6.1780498483	4.6544246419	7.4263624532
Ti	7.6086599001	4.6394695487	10.8953627893
Ti	9.0995223967	4.6039753943	14.4331843405
Ti	1.0749322768	6.5333531096	7.4470739442
Ti	2.4914843793	6.5217035350	10.9158998489
Ti	3.9089269272	6.5208164902	14.4279515759
Ti	3.8445162244	6.5331636498	8.0464070033
Ti	5.3502651082	6.5288435668	11.6675168450
Ti	6.8969783681	6.5209965828	15.1417463888
Ti	8.9442687455	4.6555224160	8.0451281297
Ti	0.2233104152	4.6375596427	11.6751989410
Ti	1.6281111038	4.6361010895	15.2489165835
O	4.2428807247	4.6544366891	8.0504698674
O	5.7225323249	4.6495938475	11.6211449578
O	7.2124161949	4.6204797552	15.2408445047

O	9.3424735639	6.5411825073	8.0074918254
O	0.6085280346	6.5350012380	11.5566468118
O	2.1855751673	6.6111991808	15.3472622655
O	2.0184286824	6.5316942453	8.9798041093
O	3.5446034506	6.5078154083	12.5179529951
O	5.1232148067	6.5038697703	16.1026114573
O	7.0996569356	4.6707587633	8.9589270061
O	8.6367813303	4.6003857876	12.4399375222
O	10.0716436979	4.7534761038	15.9728392427
O	0.5666485546	4.6545179811	7.2255908161
O	2.1033363869	4.6458128219	10.9902126992
O	3.5897993590	4.6481482088	14.6068593686
O	5.6596100137	6.5396212747	7.2080437772
O	7.1954683659	6.5358888337	10.9842331492
O	8.6203341999	6.5355433107	14.3437486294
O	2.6904792413	6.5240982698	6.6006453435
O	4.2606914501	6.5291509453	10.1111525481
O	5.7673637749	6.5814890377	13.6760862709
O	7.7912674370	4.6573944083	6.5877645335
O	9.3494945489	4.6648051435	10.0571031860
O	0.6281261484	4.6025015822	13.5730002561
Ti	6.1670106742	8.4261812747	7.4321384258
Ti	7.5920591449	8.4271372213	10.8988504809
Ti	9.0925933623	8.4392011037	14.4231814186
Ti	1.0737140211	10.3188682576	7.4451705457
Ti	2.5056243980	10.3131012220	10.9334178374
Ti	4.0163120462	10.2897120347	14.5207103145
Ti	3.8381382739	10.3187490398	8.0332303064
Ti	5.3238464456	10.3174407672	11.6109173551
Ti	6.7739778822	10.3197994420	15.0843547306
Ti	8.9421531628	8.4267444545	8.0031951595
Ti	0.2259289254	8.4202119556	11.5697158451
Ti	1.6427897448	8.4254907195	15.0848827685
O	4.2424529121	8.4247367560	8.0506060115
O	5.7333839283	8.4234754686	11.5948007498
O	7.2852878065	8.4347729403	15.2940971910
O	9.3388778796	10.3152504048	7.9974685827
O	0.6158170218	10.3071138118	11.5392755463
O	2.1848864821	10.2868239737	15.3447913868
O	2.0116472769	10.3135611413	8.9712386639
O	3.5739739814	10.2965318467	12.4800294585
O	5.1789293838	10.2760404770	15.9614940212
O	7.1075419037	8.4207589932	8.9569694970
O	8.6836910800	8.4469987184	12.4329156520
O	10.2070195340	8.3623812330	15.9110852957
O	0.5704217598	8.4243831796	7.2186465725
O	2.1197505449	8.4162595353	10.9708276854
O	3.6177020468	8.4185101663	14.5050888618
O	5.6616606461	10.3149911739	7.2102316166
O	7.2006551005	10.3104999350	10.9476012487
O	8.7123524258	10.3056984139	14.4643908634

O	2.6859199305	10.3258580364	6.5901334371
O	4.2555779763	10.3221231858	10.0637400287
O	5.8225492648	10.3323025953	13.5652625984
O	7.7760989776	8.4254189650	6.5707690804
O	9.3576234917	8.4213665764	10.0259699623
O	0.7118490748	8.4296637541	13.5402197573
O	8.0805882544	6.4403843070	17.0869268950
H	8.8084214988	5.7811886284	16.9123421420
H	8.5687232101	7.2846413471	17.1660634244
H	4.9641673493	5.8178403151	16.7697665240

2.4 Final state without H_2O on TiO_2

Lattice parameter: A 10.209915 B 11.328 C 25

Ti	6.1788779730	0.8758047770	7.4462989993
Ti	7.5913429556	0.8756103739	10.9384165369
Ti	9.0710302281	0.8771348930	14.5421106504
Ti	1.0709037971	2.7579035661	7.4484813433
Ti	2.4739515627	2.7650906823	10.9449840584
Ti	3.9222488320	2.7611517158	14.5844770404
Ti	3.8373276352	2.7630928906	8.0508978280
Ti	5.2989061792	2.7644366511	11.6497384021
Ti	6.7212039394	2.7564898333	15.0952859820
Ti	8.9432783148	0.8682948909	8.0468681535
Ti	0.2008904474	0.8705751945	11.6460081520
Ti	1.6316328651	0.8605531121	15.1282263236
O	4.2404723218	0.8764768907	8.0493398274
O	5.7018631744	0.8772799536	11.5850821954
O	7.2357320325	0.8666024174	15.3506221539
O	9.3484778496	2.7599358268	8.0440188408
O	0.5868445997	2.7539778955	11.5836108163
O	2.1428068671	2.6846193760	15.3984628794
O	2.0009131666	2.7631660674	8.9765704758
O	3.5274946682	2.8368175019	12.5044034189
O	5.1153762273	2.7488244317	15.9809920262
O	7.1129576201	0.8752616391	8.9734008389
O	8.6489160279	0.8665492231	12.4831285719
O	10.2150507401	0.8500442004	15.9759579967
O	0.5663055451	0.8668299490	7.2298775978
O	2.0937886454	0.8771757014	10.9962949620
O	3.5645090798	0.8562683124	14.5023485850
O	5.6671659842	2.7644520145	7.2239007749
O	7.1697128373	2.7592565671	10.9675090410
O	8.6572633455	2.7529804690	14.5172422874
O	2.6894340692	2.7637525293	6.6012991917
O	4.2309829165	2.7582754074	10.0868323462
O	5.7595637564	2.7366650707	13.5859616193
O	7.7926716508	0.8591791837	6.5974134675
O	9.3469265278	0.8606024799	10.0757865631
O	0.6805411785	0.8637058977	13.5911502310
Ti	6.1819883556	4.6520924326	7.4315167149
Ti	7.6056197952	4.6229416195	10.9118014956
Ti	9.0625314090	4.6574875105	14.4800840734
Ti	1.0642230730	6.5291239882	7.4128558562
Ti	2.4225282177	6.5411538681	10.8305776228
Ti	3.9882185691	6.5857440699	14.7443891322
Ti	3.8554541858	6.5365942975	8.0556531776
Ti	5.3965151116	6.5368509739	11.7159411183
Ti	6.7954651730	6.5322795051	15.2495276958
Ti	8.9439445820	4.6506361239	8.0690250555
Ti	0.2032981919	4.6429112658	11.7370932602
Ti	1.6074949430	4.6791149357	15.3269377306
O	4.2429577415	4.6586195051	8.0644281479
O	5.6915355443	4.6533944540	11.6079238110
O	7.1877196563	4.6321796678	15.2725439866
O	9.3533841088	6.5357122338	8.0459321121
O	0.5639849049	6.5393605524	11.5354470680
O	2.1869030439	6.6572240999	15.5415793915
O	1.9956786534	6.5269468405	8.9639074525
O	3.4402008734	6.2665845075	12.5903857911
O	5.1564062559	6.5932604129	16.1120662554
O	7.0951760531	4.6699598812	8.9685352356
O	8.6136958662	4.6201979830	12.4692714015
O	10.0396884418	4.7761347696	16.0146022435
O	0.5621868773	4.6542885214	7.2187342308
O	2.0657098503	4.6528019130	10.9817747471
O	3.5442657743	4.6738580435	14.6473742343
O	5.6605899886	6.5407586215	7.2196032200
O	7.1774370353	6.5408630862	11.0199753305
O	8.6026825375	6.5595464234	14.3940165288
O	2.6977686055	6.5259037782	6.6049287467
O	4.2219920750	6.5661426161	10.1608614267
O	5.7302127170	6.5776607469	13.6809318338
O	7.7964912898	4.6626383522	6.6002684091
O	9.3266331197	4.6391076412	10.0788541369
O	0.6249405216	4.6647022205	13.6116229883
Ti	6.1760405219	8.4305113148	7.4421335043
Ti	7.5896390722	8.4498125798	10.9236832788
Ti	9.0493103676	8.4308065249	14.4915543340
Ti	1.0743655730	10.3115144302	7.4546449512
Ti	2.4915896844	10.3252860055	10.9488947148
Ti	3.9658391570	10.3092401098	14.5373110644
Ti	3.8396492845	10.3197693547	8.0503326600
Ti	5.3040446088	10.3277473627	11.6408921991
Ti	6.7352354859	10.3241325429	15.1108178525
Ti	8.9447606630	8.4229276881	8.0449674007
Ti	0.2045127025	8.4227277901	11.6463633902
Ti	1.6161362275	8.4248716131	15.1398121951
O	4.2441715818	8.4243607638	8.0552373503
O	5.6930568294	8.4270529338	11.6053119363
O	7.2514592844	8.4571022293	15.3485268088
O	9.3468131709	10.3078969564	8.0426957222
O	0.6087699120	10.3057553000	11.5682704491
O	2.1545114507	10.2959834813	15.3803650705
O	2.0072273795	10.2884407463	8.9859616181
O	3.5483766301	10.3326468134	12.4890566269
O	5.1180447414	10.2836059512	15.9699994663
O	7.1059258904	8.4168892187	8.9738755255
O	8.6552306562	8.4754362022	12.4649136461
O	10.1724190108	8.4123939640	15.9627723703
O	0.5657421600	8.4193052743	7.2171202370
O	2.1286737643	8.4058827660	11.0445689057
O	3.5534628053	8.4254091468	14.4465720356
O	5.6656384301	10.3164443510	7.2250609070
O	7.1774653605	10.3170267310	10.9636258029
O	8.6637530222	10.3220577163	14.4997495486
O	2.6874611125	10.3278944175	6.6031830097
O	4.2449524020	10.3270844601	10.0762122296
O	5.7908872212	10.3271142164	13.5838125701
O	7.7917712582	8.4242574837	6.5970958508
O	9.3445439776	8.4268322343	10.0693350454
O	0.6800945415	8.3907128113	13.5866309131
O	8.0238231260	6.4453950603	17.1524430649
H	8.7609988105	5.8003117823	16.9762938341
H	8.4960184272	7.2949121036	17.2562391789
H	3.3268177793	5.3028241989	12.7625614569

3.1 Initial configuration for the isolated H atoms on TiO_2, σ=1/3

Lattice parameter: A 10.209915 B 11.328 C 25

Ti	6.0659190016	0.9353772350	7.3272077852
Ti	7.5916089961	0.9108591517	10.9611428781
Ti	8.7125685623	0.8484702180	14.4236216447
Ti	1.0629116605	2.7875741705	6.5788892537
Ti	2.5632084456	2.7802116328	10.5615474648
Ti	4.0240843322	2.7091694733	14.4805928352
Ti	3.7705527622	2.7835724616	7.6673302002
Ti	5.1637350097	2.7659360612	11.5744955570
Ti	6.7238730043	2.7311209620	15.5775875475
Ti	9.3209864341	0.8890724058	7.8803071016
Ti	0.2271668823	0.9213480692	11.3351045484
Ti	1.7555686027	0.8391623505	14.9708288988
O	4.1486276781	0.9174401121	7.8746054481
O	5.5024748243	0.8786165002	11.5830164967
O	7.2917019843	0.8913869655	15.8423066198
O	9.7240236444	2.7796470898	7.9273417434
O	0.5557764168	2.7365599765	11.1596426380
O	2.1091953253	2.6721034936	15.3891832661
O	1.9546498523	2.7751831571	8.5490954530
O	3.4772424812	2.8564182425	12.4163579071
O	4.8915375970	2.6760914815	16.3315402753
O	6.4746984057	0.9225036789	9.1624762855

O	8.9441268758	0.8110605304	12.5223016597
O	10.1857257950	0.6822587018	15.3936644608
O	0.4682536323	0.8751139775	6.5541044006
O	2.1489407548	0.8714954636	10.6263746436
O	3.6557402615	0.8163134512	14.4524979762
O	5.5964140475	2.7496441727	6.8972332180
O	7.1642961361	2.7246675469	11.3016951885
O	8.4437308625	2.7025140545	14.4402831636
O	2.8326645245	2.7681729182	6.1150739013
O	4.4481666445	2.8519698186	9.8535984250
O	5.9452679686	2.7048964998	13.7279102834
O	7.8282616568	0.9330421383	6.8922106282
O	9.2466144016	0.8727902947	9.8228427683
O	1.3830963937	0.9069612526	13.1217568806
Ti	6.0757771917	4.6321369895	7.3338738349
Ti	7.5702389505	4.6377544663	10.9440148837
Ti	8.7349234703	4.6336963409	14.4251967270
Ti	1.0700428770	6.6105483834	6.9879235386
Ti	2.5061949414	6.4769911453	10.7894840941
Ti	3.8711960276	6.5188452350	14.3711921236
Ti	3.8847579437	6.5712928430	7.7827003003
Ti	5.2900303712	6.5879443652	11.4098179586
Ti	6.6916190838	6.5424448746	15.4689203778
Ti	9.2377789112	4.6784937644	7.9547435478
Ti	0.2273122387	4.6392632987	11.4381617399
Ti	1.7301082013	4.5592827799	14.9819715080
O	4.1507665086	4.5942099627	7.7931971473
O	5.6618296719	4.6810434880	11.6873685657
O	7.1689275670	4.6097319398	15.5741735371
O	9.4454616960	6.5859363719	7.9366359046
O	0.6613310569	6.5584351731	11.4282799124
O	1.9949150554	6.4580612918	15.1701119567
O	1.9633793694	6.8689802702	8.7014057482
O	3.5566278957	6.5680083046	12.3131863641
O	4.8975012259	6.8779268527	16.1781052365
O	6.4670220284	4.4053053326	9.1372236506
O	8.7825520542	4.6954675307	12.5669189208
O	10.1886439967	4.5904995960	15.4841628320
O	0.5715542717	4.7803021054	6.8045085923
O	2.1562899823	4.6990185670	10.5471711248
O	3.6558965453	4.6042979985	14.6408557728
O	5.7644491766	6.4528710034	7.0808322491
O	7.1789709546	6.6145669326	10.8737742252
O	8.4379002647	6.5653269918	14.5497620303
O	2.6966284385	6.5700048459	6.2851810368
O	4.2769471748	6.5423407416	9.8904699074
O	5.8043735234	6.2733226952	13.6604288946
O	7.8312049474	4.5500570889	6.8389325096
O	9.1562272783	4.6550463586	9.8350174529
O	1.4011146545	4.3652265617	13.1573834030

Ti	6.1571333628	8.4407164950	7.1096278409
Ti	7.6137694126	8.4374871919	10.9164319120
Ti	8.6460648054	8.4606222143	14.4549644299
Ti	1.0965691109	10.3118460678	6.6420825138
Ti	2.5271096842	10.3343003895	10.6463611285
Ti	4.0339398569	10.2539998367	14.6235074341
Ti	3.8737692263	10.3175869896	7.7618540564
Ti	5.2317638982	10.3283997199	11.5615930573
Ti	6.8465963175	10.3218614895	16.0090483626
Ti	9.0679653029	8.4511782212	7.8602994304
Ti	0.2801292250	8.4460211981	11.4280877523
Ti	1.6431127920	8.3973861516	15.0646084878
O	4.2981085417	8.5094769331	7.8010693555
O	5.6922628038	8.4289060534	11.5433178361
O	7.2191868195	8.4379753471	15.7781197007
O	9.6482359109	10.3085590230	7.9253344164
O	0.6098633259	10.3296238876	11.1687179618
O	2.1915116190	10.3570147944	15.3743528881
O	1.9279156662	10.2297483672	8.6127858267
O	3.4262939532	10.2329955030	12.2752082383
O	5.0043540329	10.2419440895	16.1586768274
O	7.1043531326	8.8622149636	8.8702472051
O	8.8055377703	8.4433959941	12.4538049332
O	10.0122531902	8.6825008182	15.4980173494
O	0.4931360926	8.3721510574	6.7302962358
O	2.0818438050	8.4262138813	10.5987644066
O	3.6284944781	8.4578223243	14.4860839663
O	5.6636317172	10.4660019780	6.9926738618
O	7.1583335501	10.3195660873	11.0511114454
O	8.0297441516	10.3413468178	14.3675681387
O	2.8774356664	10.3174730895	6.2315988834
O	4.2601093511	10.3339477963	9.8604677038
O	5.6505307758	10.2961946294	13.4920298933
O	7.9265648999	8.4512582406	6.4814913403
O	9.2995835397	8.3954262177	9.9517728944
O	1.2857828692	8.6370960038	13.1606500641
H	2.2823454768	0.9858918579	12.7358742984
H	2.2221445903	3.8897475351	12.8197551670
H	2.1543018802	9.0038979154	12.8629582466
H	5.5790569282	0.9727558171	9.5721421619
H	5.6241983461	3.9563099513	9.4549031307
H	6.9626480007	9.8207469794	9.0208751467
H	6.7743178978	2.7006591690	13.1833056499
H	5.8496471928	5.3361939753	13.3407514727
H	6.6429497739	10.3273547923	13.6598982677
H	0.9803584900	2.8269776410	8.7674750118
H	1.8886589367	7.8154995442	9.0129187247
H	0.9613694666	10.2321600754	8.8497978349
H	4.4353091296	2.7301277943	17.1847234700
H	4.7046756606	7.7833948207	16.4861320142

3.2 Final configuration for H_2 on TiO_2, σ=1/3

Lattice parameter: A 10.209915 B 11.328 C 25

Ti	6.0649737965	0.9497009956	7.2993236598
Ti	7.5884355537	0.9342001879	10.9063305493
Ti	8.7183354273	0.9244532436	14.3775376428
Ti	1.0527857451	2.8007531651	6.5484527232
Ti	2.5717169253	2.8090425765	10.5285106284
Ti	4.0409928178	2.7647014395	14.6410478294
Ti	3.7606222596	2.7908877398	7.6041069550
Ti	5.1525781816	2.7933199302	11.5940112468
Ti	6.7457868394	2.8116840793	15.5842068763
Ti	9.3228450874	0.8981121292	7.8456830242
Ti	0.2299067414	0.9408574915	11.2956471086
Ti	1.7706386643	0.8577486790	14.8871145526
O	4.1443590433	0.9307028937	7.8345907430
O	5.5528195240	0.8997950018	11.5921834021
O	7.3314782387	0.9534208971	15.8115706043
O	9.7201739526	2.7857676346	7.8793850437
O	0.5580752375	2.7587550228	11.1342683095
O	2.1822917803	2.7235345507	15.3036174805
O	1.9585516269	2.7914977696	8.5141325346
O	3.4599721913	2.7899676177	12.4118275643
O	4.8938031888	2.7372993961	16.1738300806
O	6.4635568914	1.0322004145	9.1324122996
O	8.9364127062	0.8408230742	12.4767387992
O	10.2131199072	0.7597316231	15.3326995716
O	0.4772987755	0.8776025756	6.5302898235
O	2.1341107539	0.8952776012	10.5561099036

O	3.6936799238	0.8868066775	14.4193328393
O	5.5886096109	2.7626720704	6.8479205953
O	7.1746436373	2.7740798166	11.2533726201
O	8.4493630843	2.7753109558	14.3792849555
O	2.8219585959	2.7844405773	6.0615940628
O	4.4658855334	2.8189561097	9.8546980832
O	5.9005313970	2.7668551808	13.7226277432
O	7.8264538736	0.9242446581	6.8604868509
O	9.2538915669	0.8971127577	9.7857314128
O	1.4144479469	1.0150462645	13.0613965845
Ti	6.0705845397	4.6355969335	7.3220536089
Ti	7.5736531725	4.6418433922	10.8948044860
Ti	8.7253386378	4.7233785643	14.3821423157
Ti	1.0807566235	6.6278268321	6.9843823704
Ti	2.5112127240	6.5031238768	10.8137203760
Ti	3.9314309454	6.5920331133	14.4566161948
Ti	3.8865454442	6.5773291361	7.7956565557
Ti	5.2899813565	6.5904155712	11.4236986268
Ti	6.6601528801	6.6179455832	15.4980678742
Ti	9.2526623848	4.6975522709	7.9406283646
Ti	0.2174535255	4.6492409505	11.4189072504
Ti	1.7478786178	4.6218317088	14.9572944706
O	4.1389942243	4.5931803611	7.7973240337
O	5.5969908608	4.6923960587	11.6943627010
O	7.2055052743	4.6767935409	15.5627160512
O	9.4479645693	6.6062040759	7.9134669150

O	0.6645977180	6.5826517288	11.4468320351
O	2.0947584199	6.5118191995	15.1198390364
O	1.9602905636	6.8857708238	8.7092071010
O	3.5597207088	6.5961334577	12.3489516018
O	4.8764052062	6.6435289533	15.9987062454
O	6.4412417388	4.4230817161	9.1356626766
O	8.7700016579	4.7542944594	12.5382115752
O	10.2218451947	4.6790245673	15.4205115654
O	0.5888215967	4.8009292646	6.7872435517
O	2.1745829804	4.7208197214	10.5514475372
O	3.6763539864	4.6427372816	14.4953418774
O	5.7575948486	6.4540390819	7.0720192032
O	7.1712467356	6.6037389590	10.8261634841
O	8.4322584587	6.6403055100	14.4979240606
O	2.7131686171	6.5890620441	6.2926836718
O	4.2656855368	6.5669193071	9.9088872434
O	5.7946539987	6.3154033852	13.6283951566
O	7.8189724318	4.5575085619	6.8473600558
O	9.2153770820	4.6760034296	9.7896725061
O	1.4001593738	4.3902217228	13.1430449150
Ti	6.1563006761	8.4393543099	7.0889094864
Ti	7.6156994716	8.4434758374	10.9073414973
Ti	8.6561557656	8.5177547241	14.4550653084
Ti	1.1110915483	10.3170382994	6.6317678683
Ti	2.5235999026	10.3782391368	10.6433923803
Ti	4.0644961156	10.3188143215	14.5319879155
Ti	3.8818805293	10.3236854051	7.7578859205
Ti	5.2495131277	10.3696924083	11.5066350789
Ti	6.8581740661	10.3993467655	15.9856946305
Ti	9.0670970629	8.4603326996	7.8399061514
Ti	0.2826516216	8.4572427291	11.4155138549
Ti	1.6567144544	8.4574522633	15.0529717363
O	4.3057977263	8.5158873894	7.8091260691
O	5.7206502699	8.4444350547	11.5261048794
O	7.2615766613	8.5053382990	15.7866022575
O	9.6557380934	10.3172876181	7.9130175171
O	0.6140126556	10.3577980940	11.1678491010
O	2.2140229966	10.4062412705	15.2621975679
O	1.9339621599	10.2225150030	8.6084803143
O	3.4477033293	10.2765822953	12.2671414479
O	5.0099478065	10.3657321821	16.0523709093
O	7.1110787989	8.8349149708	8.8489134455
O	8.7837010673	8.4787956168	12.4339515247
O	10.0489800703	8.7078753545	15.4992140989
O	0.4997488364	8.3847561614	6.7151970450
O	2.0845856515	8.4507672543	10.6140308049
O	3.6394457704	8.4610035410	14.4420124466
O	5.6751746442	10.4698892752	7.0031627699
O	7.1663012812	10.3379226135	10.9599600511
O	8.0532674216	10.3994057663	14.3472760474
O	2.8921382292	10.3142012201	6.2243521564
O	4.2580852363	10.3639212481	9.8471933323
O	5.6856190588	10.3010107036	13.4399925904
O	7.9262859327	8.4646069855	6.4605568516
O	9.3043194403	8.4068754381	9.9449254863
O	1.2530382346	8.7032986761	13.1591234886
H	2.2708504249	1.3873409188	12.7197931481
H	2.2331460519	3.9482401109	12.8033326823
H	2.0896231594	9.1351766987	12.8597778435
H	5.5785003926	1.2680325822	9.5095304051
H	5.5889350899	4.0012735667	9.4597447311
H	6.9655014724	9.7912101019	9.0099675814
H	6.7635958732	2.7642639437	13.2341077342
H	5.8371339473	5.3678739023	13.3322988678
H	6.6739682420	10.3618006341	13.6250354205
H	0.9865792799	2.8238834493	8.7390181274
H	1.8934575720	7.8360579440	9.0040975514
H	0.9633952460	10.2356492349	8.8309848920
H	2.6559038403	4.3294374830	17.2190921550
H	2.6037309823	5.0851097676	17.1984032404

3.3 Initial configuration with the isolated H atoms inside TiO_2, σ=1/3

Lattice parameter: A 10.209915 B 11.328 C 25

Ti	6.0659190016	0.9353772350	7.3272077852
Ti	7.5916089961	0.9108591517	10.9611428781
Ti	8.7125685623	0.8484702180	14.4236216447
Ti	1.0629116605	2.7875741705	6.5788892537
Ti	2.5632084456	2.7802116328	10.5615474648
Ti	4.0240843322	2.7091694733	14.4805928352
Ti	3.7705527622	2.7835724616	7.6673302002
Ti	5.1637350097	2.7659360612	11.5744955570
Ti	6.7238730043	2.7311209620	15.5775875475
Ti	9.3209864341	0.8890724058	7.8803071016
Ti	0.2271668823	0.9213480692	11.3351045484
Ti	1.7555686027	0.8391623505	14.9708288988
O	4.1486276781	0.9174401121	7.8746054481
O	5.5024748243	0.8786165002	11.5830164967
O	7.2917019843	0.8913869655	15.8423066198
O	9.7240236444	2.7796470898	7.9273417434
O	0.5557764168	2.7365599765	11.1596426380
O	2.1091953253	2.6721034936	15.3891832661
O	1.9546498523	2.7751831571	8.5490954530
O	3.4772424812	2.8564182425	12.4163579071
O	4.8915375970	2.6760914815	16.3315402753
O	6.4746984057	0.9225036789	9.1624762855
O	8.9441268758	0.8110605304	12.5223016597
O	10.1857257950	0.6822587018	15.3936644608
O	0.4682536323	0.8751139775	6.5541044006
O	2.1489407548	0.8714954636	10.6263746436
O	3.6557402615	0.8163134512	14.4524979762
O	5.5964140475	2.7496441727	6.8972332180
O	7.1642961361	2.7246675469	11.3016951885
O	8.4437308625	2.7025140545	14.4402831636
O	2.8326645245	2.7681729182	6.1150739013
O	4.4481666445	2.8519698186	9.8535984250
O	5.9452679686	2.7048964998	13.7279102834
O	7.8282616568	0.9330421383	6.8922106282
O	9.2466144016	0.8727902947	9.8228427683
O	1.3830963937	0.9069612526	13.1217568806
Ti	6.0757771917	4.6321369895	7.3338738349
Ti	7.5702389505	4.6377544663	10.9440148837
Ti	8.7349234703	4.6336963409	14.4251967270
Ti	1.0700428770	6.6105483834	6.9879235386
Ti	2.5061949414	6.4769911453	10.7894840941
Ti	3.8711960276	6.5188452350	14.3711921236
Ti	3.8847579437	6.5712928430	7.7827003003
Ti	5.2900303712	6.5879443652	11.4098179586
Ti	6.6916190838	6.5424448746	15.4689203778
Ti	9.2377789112	4.6784937644	7.9547435478
Ti	0.2273122387	4.6392632987	11.4381617399
Ti	1.7301082013	4.5592827799	14.9819715080
O	4.1507665086	4.5942099627	7.7931971473
O	5.6618296719	4.6810434880	11.6873685657
O	7.1689275670	4.6097319398	15.5741735371
O	9.4454616960	6.5859363719	7.9366359046
O	0.6613310569	6.5584351731	11.4282799124
O	1.9949150554	6.4580612918	15.1701119567
O	1.9633793694	6.8689802702	8.7014057482
O	3.5566278957	6.5680083046	12.3131863641
O	4.8975012259	6.8779268527	16.1781052365
O	6.4670220284	4.4053053326	9.1372236506
O	8.7825520542	4.6954675307	12.5669189208
O	10.1886439967	4.5904995960	15.4841628320
O	0.5715542717	4.7803021054	6.8045085923
O	2.1562899823	4.6990185670	10.5471711248
O	3.6558965453	4.6042979985	14.6408557728
O	5.7644491766	6.4528710034	7.0808322491
O	7.1789709546	6.6145669326	10.8737742252
O	8.4379002647	6.5653269918	14.5497620303
O	2.6966284385	6.5700048459	6.2851810368
O	4.2769471748	6.5423407416	9.8904699074
O	5.8043735234	6.2733226952	13.6604288946
O	7.8312049474	4.5500570889	6.8389325096
O	9.1562272783	4.6550463586	9.8350174529
O	1.4011146545	4.3652265617	13.1573834030
Ti	6.1571333628	8.4407164950	7.1096278409
Ti	7.6137694126	8.4374871919	10.9164319120
Ti	8.6460648054	8.4606222143	14.4549644299
Ti	1.0965691109	10.3118460678	6.6420825138
Ti	2.5271096842	10.3343003895	10.6463611285
Ti	4.0339398569	10.2539998367	14.6235074341
Ti	3.8737692263	10.3175869896	7.7618540564
Ti	5.2317638982	10.3283997199	11.5615930573
Ti	6.8465963175	10.3218614895	16.0090483626
Ti	9.0679653029	8.4511782212	7.8602994304

Ti	0.2801292250	8.4460211981	11.4280877523
Ti	1.6431127920	8.3973861516	15.0646084878
O	4.2981085417	8.5094769331	7.8010693555
O	5.6922628038	8.4289060534	11.5433178361
O	7.2191868195	8.4379753471	15.7781197007
O	9.6482359109	10.3085590230	7.9253344164
O	0.6098633259	10.3296238876	11.1687179618
O	2.1915116190	10.3570147944	15.3743528881
O	1.9279156662	10.2297483672	8.6127858267
O	3.4262939532	10.2329955030	12.2752082383
O	5.0043540329	10.2419440895	16.1586768274
O	7.1043531326	8.8622149636	8.8702472051
O	8.8055377703	8.4433959941	12.4538049332
O	10.0122531902	8.6825008182	15.4980173494
O	0.4931360926	8.3721510574	6.7302962358
O	2.0818438050	8.4262138813	10.5987644066
O	3.6284944781	8.4578223243	14.4860839663
O	5.6636317172	10.4660019780	6.9926738618
O	7.1583335501	10.3195660873	11.0511114454
O	8.0297441516	10.3413468178	14.3675681387
O	2.8774356664	10.3174730895	6.2315988834
O	4.2601093511	10.3339477963	9.8604677038
O	5.6505307758	10.2961946294	13.4920298933
O	7.9265648999	8.4512582406	6.4814913403
O	9.2995835397	8.3954262177	9.9517728944
O	1.2857828692	8.6370960038	13.1606500641
H	2.2823454768	0.9858918579	12.7358742984
H	2.2221445903	3.8897475351	12.8197551670
H	2.1543018802	9.0038979154	12.8629582466
H	5.5790569282	0.9727558171	9.5721421619
H	5.6241983461	3.9563099513	9.4549031307
H	6.9626480007	9.8207469794	9.0208751467
H	6.7743178978	2.7006591690	13.1833056499
H	5.8496471928	5.3361939753	13.3407514727
H	6.6429497739	10.3273547923	13.6598982677
H	0.9803584900	2.8269776410	8.7674750118
H	1.8886589367	7.8154995442	9.0129187247
H	0.9613694666	10.2321600754	8.8497978349
H	4.4353091296	2.7301277943	17.1847234700
H	4.7046756606	7.7833948207	16.4861320142

3.4 Final configuration for H_2 inside TiO_2, σ=1/3

Lattice parameter: A 10.209915 B 11.328 C 25

Ti	6.0053889656	0.8637619378	7.2585524687
Ti	7.6053737459	0.8413315381	10.8781241051
Ti	8.8009130329	0.8316105395	14.3766308528
Ti	0.9959131579	2.7678824706	6.6431644536
Ti	2.4903034989	2.7882808291	10.7221258810
Ti	3.9525526295	2.7692238201	14.4390908939
Ti	3.7088172724	2.7023397106	7.7023406967
Ti	5.2442743159	2.6617925472	11.5521203485
Ti	6.8595838810	2.7903131382	15.4058110132
Ti	9.2458261260	0.8410730134	7.8440840991
Ti	0.2089145534	0.8287216944	11.3269654743
Ti	1.7817146249	0.8411579285	14.9683546497
O	4.1137032628	0.8482683018	7.9186195716
O	5.6259174828	0.8344012176	11.6126131995
O	7.2768353289	0.8698665783	15.5485889625
O	9.6390381004	2.7390108518	7.9149129995
O	0.5537142136	2.6984155166	11.2278582435
O	2.0709522266	2.6877465030	15.3237450610
O	1.9140458490	2.7574369955	8.6443665621
O	3.4938658270	2.6215367729	12.3362051110
O	5.0072959613	2.6817064390	16.1815794891
O	6.4479455225	0.8469430918	9.0934643628
O	8.8859817761	0.7973471354	12.4655177081
O	10.2397294341	0.6392206605	15.4297170135
O	0.4494818487	0.8313518443	6.6087970326
O	2.0802317418	0.8203251488	10.6346952514
O	3.6941696076	0.8107249067	14.4853703090
O	5.5063362542	2.6794996310	6.8560361155
O	7.1693402386	2.7083619527	10.9942335684
O	8.6656470536	2.7214728338	14.4365023535
O	2.7616608879	2.6929722717	6.1673012738
O	4.2866570671	2.7690601353	9.9321444798
O	5.8488586615	3.0476421959	13.6206417220
O	7.7589883139	0.9148473315	6.8200087051
O	9.2914099199	0.8257135580	9.7837666642
O	1.3646616429	0.9104767425	13.1365602482
Ti	5.9899165498	4.5650007783	7.2418181457
Ti	7.5533347944	4.5700736320	10.9239977337
Ti	9.1017808398	4.6184003518	14.4706346000
Ti	0.9962302008	6.5909036803	7.0663657571
Ti	2.4157485591	6.4797649360	10.9078338345
Ti	4.0627209378	6.5065399000	14.3627653845
Ti	3.8074048324	6.5008024242	7.7740312294
Ti	5.2567140493	6.4930057639	11.1945928130
Ti	6.9500626683	6.5084930258	15.5760254909
Ti	9.1488299107	4.6379071530	7.9361853583
Ti	0.1168499547	4.5682984500	11.5009707581
Ti	1.6476265371	4.6380085621	15.0446255170
O	4.0983354438	4.5111262195	7.8203866202
O	5.6867962315	4.6155519142	11.5887758794
O	7.4098359688	4.6095332398	15.6032468354
O	9.3344694425	6.5415166899	7.9439922240
O	0.4024402878	6.5190324856	11.2129059705
O	2.0755172285	6.5352413332	15.2941161482
O	1.8942365547	6.9410079995	8.7554561444
O	3.8375244946	6.5096103990	12.3014502720
O	5.0295626937	6.5250735102	16.1776019103
O	6.4094993391	4.4614079530	9.0734347684
O	8.5457324489	4.6650847919	12.4663173001
O	10.2527837195	4.6162282920	15.9154148582
O	0.5143863756	4.7503199730	6.8633129840
O	2.0845224315	4.5688379944	10.7963643889
O	3.5426357882	4.6328123592	14.4617365382
O	5.6519545621	6.3651908959	6.9378314552
O	7.2012796311	6.4995650497	10.7768864228
O	8.7402728926	6.5316118662	14.4903673070
O	2.5972882112	6.5122832856	6.3053760937
O	4.1631030046	6.4160103055	9.7919600974
O	6.0770132846	6.5051213600	13.8142511284
O	7.7558993165	4.4779044978	6.8003673128
O	9.0915432266	4.5653943452	9.8186620321
O	0.6549114467	4.5745614624	13.5132303507
Ti	6.0773973560	8.3518514364	7.0018610012
Ti	7.6013675540	8.3696081810	10.8298490820
Ti	9.0629987295	8.3948902672	14.4766918485
Ti	1.0381639123	10.2543247559	6.7429447893
Ti	2.4969965899	10.2710718787	10.8229632953
Ti	4.0504716242	10.2358931979	14.4609510533
Ti	3.8431163171	10.2390367514	7.8357718353
Ti	5.2885718303	10.2419965367	11.4977143936
Ti	6.9419386535	10.2147348605	15.8267666331
Ti	8.9602164335	8.4089262496	7.8073552331
Ti	0.1676254895	8.3649530576	11.4866941690
Ti	1.6584888300	8.3825550943	14.9642787447
O	4.2664943713	8.4333191259	7.8283723463
O	5.6766686720	8.3671610204	11.3324673166
O	7.4713031673	8.3600642225	15.7991584632
O	9.4973623429	10.2689274417	7.8503844007
O	0.5496867261	10.2869844965	11.2767780885
O	2.2217512396	10.2770212462	15.2387815340
O	1.8344833007	9.9734102009	8.7180389136
O	3.5458471454	10.3192998104	12.3587512755
O	5.0980571041	10.2041398416	15.9449817218
O	7.0447024764	8.7534316326	8.7571080167
O	8.5906821335	8.3552760396	12.3795765787
O	10.2699037200	8.5273280872	15.8548269119
O	0.4141883937	8.3241430522	6.7337802965
O	2.0569925066	8.4237618793	10.9572672195
O	3.5639214189	8.4013567921	14.3778242474
O	5.5936298178	10.3901640242	6.9704875511
O	7.1664702993	10.2708247280	10.9024109584
O	8.3249491421	10.2232371223	14.3743530564
O	2.8205924039	10.2745481381	6.3358145629
O	4.1867759865	10.2946911026	9.9003964256
O	5.7934969241	10.1297715655	13.3993035933
O	7.8499010329	8.3635541726	6.3932795791
O	9.2692840555	8.4550870670	9.9083104779
O	0.5996342544	8.4638376248	13.4607597470
H	2.2479526566	1.1327016772	12.7567148150
H	1.4973214719	6.2702417918	12.9048887792
H	2.0823495927	6.7288126166	12.9499200729
H	5.5500317124	0.8881474536	9.4936521911

H	5.5373510177	4.1719984956	9.4371356916
H	6.9070599190	9.7097486652	8.9273115733
H	6.0000712359	3.9866910693	13.3549064328
H	6.8212859189	6.5526544638	13.1834371142
H	6.7775972143	10.0821434399	13.5144580490
H	0.9434006017	2.7290872452	8.8541348165
H	1.8681887542	7.9184479556	8.9135230109
H	0.9090299367	10.0880961845	9.0357636619
H	4.6877269645	2.2930602925	17.0103808690
H	4.5961946630	6.5839789039	17.0427143302

Supplementary References

1. Egerton, R. F., McLeod, R., Wang, F. & Malac, M. Basic Questions Related to Electron-induced Sputtering in the TEM. *Ultramicroscopy* **110**, 991–997 (2010).
2. Schatz, T., Cook, A. R., & Meisel, D. Charge carrier transfer across the silica nanoparticle/water interface. *J. Phys. Chem. B,* **102**, 7225-7230 (1998).
3. Le Caër, S. Water radiolysis: influence of oxide surfaces on H_2 production under ionizing radiation. *Water* **3**, 235-253 (2011).
4. Chaplin, M. *Water structure and science*. (2011).
5. Schneider, *et al*. Electron–water interactions and implications for liquid cell electron microscopy. *J. Phys. Chem. C* **118**, 22373-22382 (2014).
6. Grogan, J. M., Schneider, N. M., Ross, F. M., & Bau, H. H. Bubble and pattern formation in liquid induced by an electron beam. *Nano letters* **14**, 359-364 (2013).
7. Buckett, M. I., et al. Electron Irradiation Damage in Oxides. *Ultramicroscopy* **29**, 217−227 (1989).

8 Knotek, M. L., Feibelman, P. J. Stability of Ionically Bonded Surfaces in Ionizing Environments. *Surf. Sci*. **90**, 78−90 (1979).

9. Selcuk, S., & Selloni, A. Facet-dependent trapping and dynamics of excess electrons at anatase TiO_2 surfaces and aqueous interfaces. *Nat. Mater*. **15**, 1107-1112 (2016).
10. Ji, Y., Wang, B. & Luo, Y. A Comparative Theoretical Study of Proton-Coupled Hole Transfer for H_2O and Small Organic Molecules (CH_3OH, HCOOH, H_2CO) on the Anatase TiO_2(101) Surface. *J. Phys. Chem. C* **118**, 21457-21462 (2014).
11. Setvin, M. *et al.* Direct view at excess electrons in TiO_2 rutile and anatase. *Phys. Rev. Lett.* **113**, 086402 (2014).
12. Scanlon, D. O. *et al.* Band alignment of rutile and anatase TiO_2. *Nat. Mater.* **12**, 798-801, (2013).
13. Liu, L., Yu, P. Y., Chen, X., Mao, S. S. & Shen, D. Z. Hydrogenation and disorder in engineered black TiO_2. *Phys. Rev. Lett.* **111**, 065505 (2013).
