# Supplementary material for: Self-hydrogenated shell promoting photocatalytic H2 evolution on anatase TiO2
Source: Nat Commun. 2018 Jul 16;9:2752. doi: 10.1038/s41467-018-05144-1 (PMC6048119; doi:10.1038/s41467-018-05144-1)
Supplement: Supplementary file 2 — Description of Additional Supplementary Files [file 41467_2018_5144_MOESM2_ESM.pdf]

Description of Additional Supplementary Files

File Name: Supplementary Movie 1
Description: FPMD calculations of the water molecules dissociation on the hydrogenated TiO_2 surface.

File Name: Supplementary Movie 2
Description: FPMD calculations of the water formation inside the hydrogenated TiO_2 slab.
